# Learning from urban form to predict building heights

**DOI:** 10.1371/journal.pone.0242010

**Published:** 2020-12-09

**Authors:** Nikola Milojevic-Dupont, Nicolai Hans, Lynn H. Kaack, Marius Zumwald, François Andrieux, Daniel de Barros Soares, Steffen Lohrey, Peter-Paul Pichler, Felix Creutzig

**Affiliations:** 1 Chair of Sustainability Economics, School of Planning, Building and Environment, Technische Universität Berlin, Berlin, Germany; 2 Working group Land Use, Infrastructure and Transport, Mercator Research Institute on Global Commons and Climate Change (MCC), Berlin, Germany; 3 Applied Statistics, School of Business and Economics, Humboldt-Universität zu Berlin, Berlin, Germany; 4 Department of Humanities, Energy Politics Group, Social and Political Sciences, ETH Zürich, Zürich, Switzerland; 5 Department of Environmental Systems Science, Weather and Climate Risk Group, ETH Zürich, Zürich, Switzerland; 6 Climate Physics Group, Department of Environmental Systems Science, ETH Zürich, Zürich, Switzerland; 7 Nam.R, Paris, France; 8 FutureLab Social Metabolism and Impacts, Potsdam Institute for Climate Impact Research (PIK), Potsdam, Germany; National University of Singapore, SINGAPORE

## Abstract

Understanding cities as complex systems, sustainable urban planning depends on reliable high-resolution data, for example of the building stock to upscale region-wide retrofit policies. For some cities and regions, these data exist in detailed 3D models based on real-world measurements. However, they are still expensive to build and maintain, a significant challenge, especially for small and medium-sized cities that are home to the majority of the European population. New methods are needed to estimate relevant building stock characteristics reliably and cost-effectively. Here, we present a machine learning based method for predicting building heights, which is based only on open-access geospatial data on urban form, such as building footprints and street networks. The method allows to predict building heights for regions where no dedicated 3D models exist currently. We train our model using building data from four European countries (France, Italy, the Netherlands, and Germany) and find that the morphology of the urban fabric surrounding a given building is highly predictive of the height of the building. A test on the German state of Brandenburg shows that our model predicts building heights with an average error well below the typical floor height (about 2.5 m), without having access to training data from Germany. Furthermore, we show that even a small amount of local height data obtained by citizens substantially improves the prediction accuracy. Our results illustrate the possibility of predicting missing data on urban infrastructure; they also underline the value of open government data and volunteered geographic information for scientific applications, such as contextual but scalable strategies to mitigate climate change.

## Introduction

Urban planners are appointed to improve the quality of life in their jurisdictions, and face the double challenge of mitigating climate change and adapting to its inevitable consequences [1–5]. To design urban planning strategies and efficiently allocate limited resources, policy makers need accurate and comprehensive data on urban infrastructures. In addition to standard tools, such as cadaster maps and building codes, planners and researchers would profit from novel planning instruments such as material stock models and 3D models of urban form that provide high-resolution data for upscaling contextually rich climate solutions [1, 6].

In this context, building height information is of increasing relevance to urban planning, serving as a key input to urban climate models [7], analyses of sprawl [8], resilience planning [9], and many other planning approaches [10]. The height information in 3D building models can have several levels of precision, which range from a single height per building (sometimes called 2.5D model) to detailed textured 3D models with photo-realistic representations of facades and roof details. Different applications come with different requirements on resolution but often a single building height at precision level corresponding to the number of floors (approximately 2.5 m, see details in Training and evaluation) serve planners’ purposes. One use case of 3D building models is to support efforts to reduce energy use in buildings, where they are key to constructing ‘Urban Building Energy Models’ [11]—models that simulate building energy use consistently across neighborhoods and cities [12–16]. Precise height information for example allows to account for the impact of the surrounding buildings on a building’s thermal performance [17]. Simpler information such as approximate building heights, volume or floor space are useful for downscaling energy use models to obtain regional estimates [18], studying environmental impacts of buildings [19] and would be valuable in global models of urban density such as [20].

3D building models are mainly generated for public authorities and large technology companies, and not widely available to researchers, small public entities and citizens. However, for open governance processes it is crucial that these three user groups have access to such data. Generating detailed 3D building models is costly, as it requires high-quality remote sensing imagery and complex post-processing. This results in large gaps in the spatial coverage of these models, particularly for smaller cities and lower-income countries. In Europe, cities (e.g. Vienna or Helsinki), regions (e.g. Nordrhein-Westfalen or Brandenburg), and countries (e.g. France, the United Kingdom and Germany) have acquired a 3D models of their building stock, often through combining of aerial sensing (LiDAR) and their own cadaster data. But currently few cities, regions and countries—e.g. the Netherlands [21]—make this data freely available to all. In most cases, the data need to be bought or obtained under specific conditions. Furthermore, open datasets are scattered over various jurisdictions’ websites in various formats, which constitutes a technical barrier for users. Large technology companies including Google and Microsoft, and start-ups such as Blackshark.ai are also developing high-quality 3D models covering large regions. They use photogrammetry and state-of-the-art computer vision approaches on proprietary data including large amounts of satellite imagery and existing map information, which are often inaccessible for external analysts. While the 3D data are publicly displayed on map products such as Google Maps or Bing, the access to bulk data for scientific and public activities is limited.

The contribution of this study is to explore how publicly available data and machine learning techniques can help fill gaps in 3D building data over large areas with no or few administrative data available. Here, we use machine learning techniques, domain knowledge from urban studies, and data from volunteered geographic information (VGI) to extend the spatial coverage of openly available 3D models. Specifically, we use urban morphology features to train a gradient boosting algorithm to predict building heights across four European countries (France, Germany, Italy and the Netherlands). Our analysis expands on an earlier proof of concept [22], by using a larger dataset with more spatial diversity, and a set of predictors that is more widely available. We are particularly interested here in assessing (i) how the model generalizes to unseen data from another country, (ii) how the model performs across lower and taller buildings, (iii) how the performance can be enhanced by adding local data to the training set—either randomly sampled data points (similar to availability in OpenStreetMap) or data on the main city of a region (which is more likely to have a 3D model). We also compare the importance of different urban form features for the prediction.

For this, first we create a wide set of urban form features. In Materials and Methods, we describe the urban morphology data, various features created based on the data and the prediction models used. In Experiments, we perform a cross-country validation for model selection and to investigate the model’s ability to generalize across areas. We then illustrate three experiments conducted to evaluate the model performance with respect to the research questions. We address these research questions in detail in Results. In the Discussion section, we elaborate on how our proof-of-concept could be extended to make open infrastructure data available at the European scale and discuss implications for policy makers.

## Materials and methods

We train a supervised machine learning model to predict building heights based only on publicly accessible urban form data. Supervised learning is well-suited to extend the spatial coverage of limited ground truth measurements [12, 13], and has been successfully applied to predicting building heights [22] and various other building attributes e.g. building ages [23, 24]. A previous study has used tree-based ensemble methods to predict building heights in two Dutch cities [22]. The best models of this study achieve good performance with a mean absolute error below one meter using little training data (20,000 buildings). As they use cadaster data as predictors, in particular the number of floors of a building—whose availability is limited—this approach has a limited scalability. We build on this previous study by using a larger dataset that includes several countries, and only rely on urban morphology predictors that are available at scale. The morphology of neighborhoods and road networks has been described quantitatively in the fields of urban science [25–27]. These studies developed a quantitative understanding of urban form, based, for example, on complex network analysis, and provide metrics from which machine learning algorithms can learn. Lastly, our workflow builds upon open-source software for geospatial analysis [28–30] and machine learning [47, 53].

We predict building heights for a large test area in Germany. To train and validate, we use data on building heights and urban form from several areas across Europe. We use domain knowledge to generate 152 features that describe the building’s footprint geometry and its surroundings. We compare the performance of several learning algorithms for this prediction task. In this section, we describe the data, feature engineering, and learning algorithms used.

### Data

**Fig 1:**
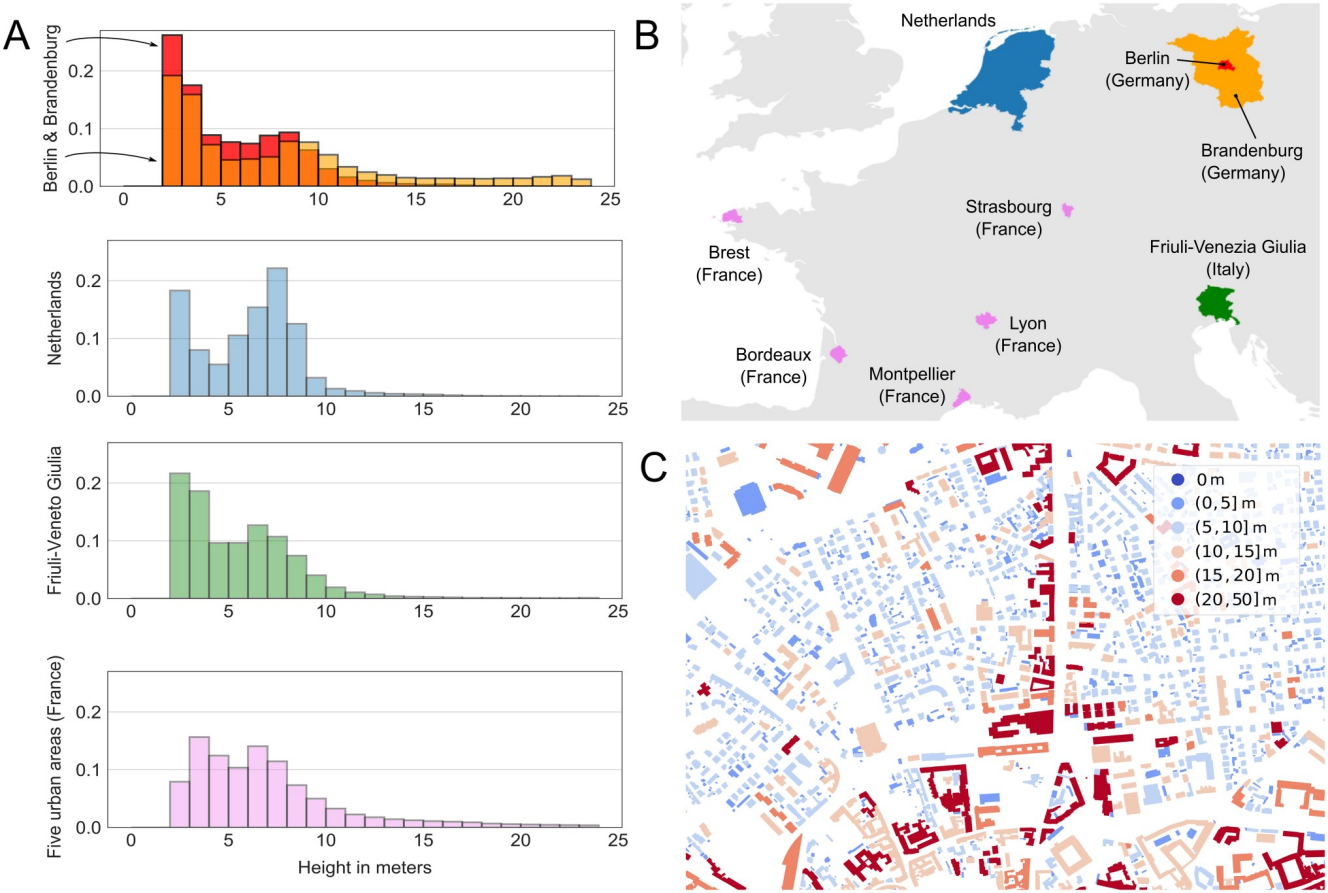
Building heights in the different geographical areas used in this study. (**A**) Height distributions for the state of Brandenburg in Germany, the Netherlands, the region of Friuli-Venezia Giulia in Italy and five French urban areas. These distributions correspond to the final dataset, after removing buildings with a height below 2 m and buildings with a footprint area below 10 m^2^, see S4 Appendix. (**B**) Location of the four areas representing Northern and Southern European regions, and urban and rural contexts. (**C**) Example of building heights in the city of Udine in Italy. This map shows a mid-size city with higher buildings in the historical center and along a main axis, and lower buildings in residential areas.

Our dataset includes ∼11.5 million buildings across 920 cities and four European countries—Germany, Netherlands, France and Italy. We used only open data, from either government sources or in form of VGI. OpenStreetMap (OSM), such a VGI initiative, provides urban form data in Europe with a good to very good coverage for roads [31] and buildings [32, 33], depending on the region. This section provides a overview of the datasets used. We report key descriptive statistics in Fig 1 and Table 1, and more detailed information about the pre-processing of the data in S1 Appendix.

**Table 1:** Summary statistics of the full dataset.

Country	Region	# buildings	Mean h (m)	Median h (m)	Standard dev* (m)	Cities 0 − 5k	Cities 5 − 20k	Cities 20 − 50k	Cities 50k+
*Germany*	Brandenburg	2,127,956	5.64	4.68	3.44	265	133	13	0
*Germany*	Berlin	509,472	8.56	6.93	6.69	0	0	0	1
*Netherlands*	All twelve regions	7,674,787	6.18	6.52	2.75	35	142	45	11
*Italy*	Friuli-Venezia Giulia	534,483	5.11	5	3.3	194	19	2	0
*France*	Five urban areas	654,881	7.42	6.35	5.03	15	38	6	1
Total		11,465,176	6.21	6.23	3.41	509	332	66	14

These counts correspond to the final cleaned dataset. The five last columns are the counts of cities in each area that contain a given number of buildings, e.g. between 5,000 and 20,000 buildings. References: Brandenburg [34], Berlin [35], the Netherlands [36], Friuli-Venezia Giulia [37], and for the five French urban areas, Bordeaux [38], Brest [39], Montpellier [40], Lyon [41] and Strasbourg [42].

*Standard Dev: Standard deviation.

Our dataset represents a diversity of geographical settings, such as Northern and Southern European, as well as urban and rural regions. Five French urban areas (the cities of Bordeaux, Brest, Montpellier, Lyon and Strasbourg and their surroundings) and Berlin in Germany are urbanized areas with a higher proportion of taller buildings. In contrast, Friuli-Venezia Giulia in Italy is a rural area: more than 90% of the cities have less than 5,000 buildings and there is a higher proportion of small buildings. The Netherlands and Brandenburg in Germany are areas with small- and medium-sized cities and rural areas. Most data are from the Netherlands (about 7.7 million buildings).

#### Building heights

Our target variable is a single height value for each building. Many buildings have ambiguous heights, for example because some building and roof parts are higher than others. For a detailed discussion on how to define building heights, see [10]. The various data sources in our dataset define the height of a building differently (e.g. highest point of the roof, highest point of the walls, etc.). For example, in the Netherlands, heights are provided as a percentile of the point cloud from the radar scanning of the building, while for Friuli-Venezia Giulia there is no information available. When several height values were available, we chose values most consistent across regions. We use *h* to denote the ‘ground truth’ height of a building.

We extract height information from 3D models covering all buildings in the Netherlands, the regions of Berlin and Brandenburg, and five urban areas in France. We complement these data with height data available in OpenStreetMap for more than 90% buildings in the region of Friuli-Venezia Giulia in Italy. Some of these files provide the height of the building as a value. Others contain detailed geometries, and we extracted the highest point of each building geometry, see S1 Appendix for details.

The distributions of building heights in the five areas show both similarities and clear differences (Fig 1A). Most of the mass of the distribution is between 2 and 10 meters, and the median values are around 6 meters. Distributions tend to be bi-modal, with peaks for single-story buildings (2–3 meters) and for buildings with two or three floors (7–9 meters). The amplitude of the peaks, however, varies substantially between the regions with some cities having a much larger share of higher buildings than others. Depending on the region, we observe some spatial auto-correlation of building heights. For example, similar heights are relatively more clustered in Berlin, but less so in Brandenburg (see analysis in S4 Table).

#### Urban morphology data

To create the features for our model, we extract data on the urban morphology such as buildings’ geometries from the same datasets that contain the height information, and OpenStreetMap. For each building, we either extract the building footprint from a 3D dataset where available or match it with the building’s footprint in OpenStreetMap. The footprint is used to geolocate the building and compute its features.

Street networks are exclusively retrieved from OpenStreetMap. Some preprocessing is done to clean the streets (e.g. multiple linestrings for two-way streets) and to generate block-based streets by turning street linestrings into polygons.

### Feature engineering

**Fig 2:**
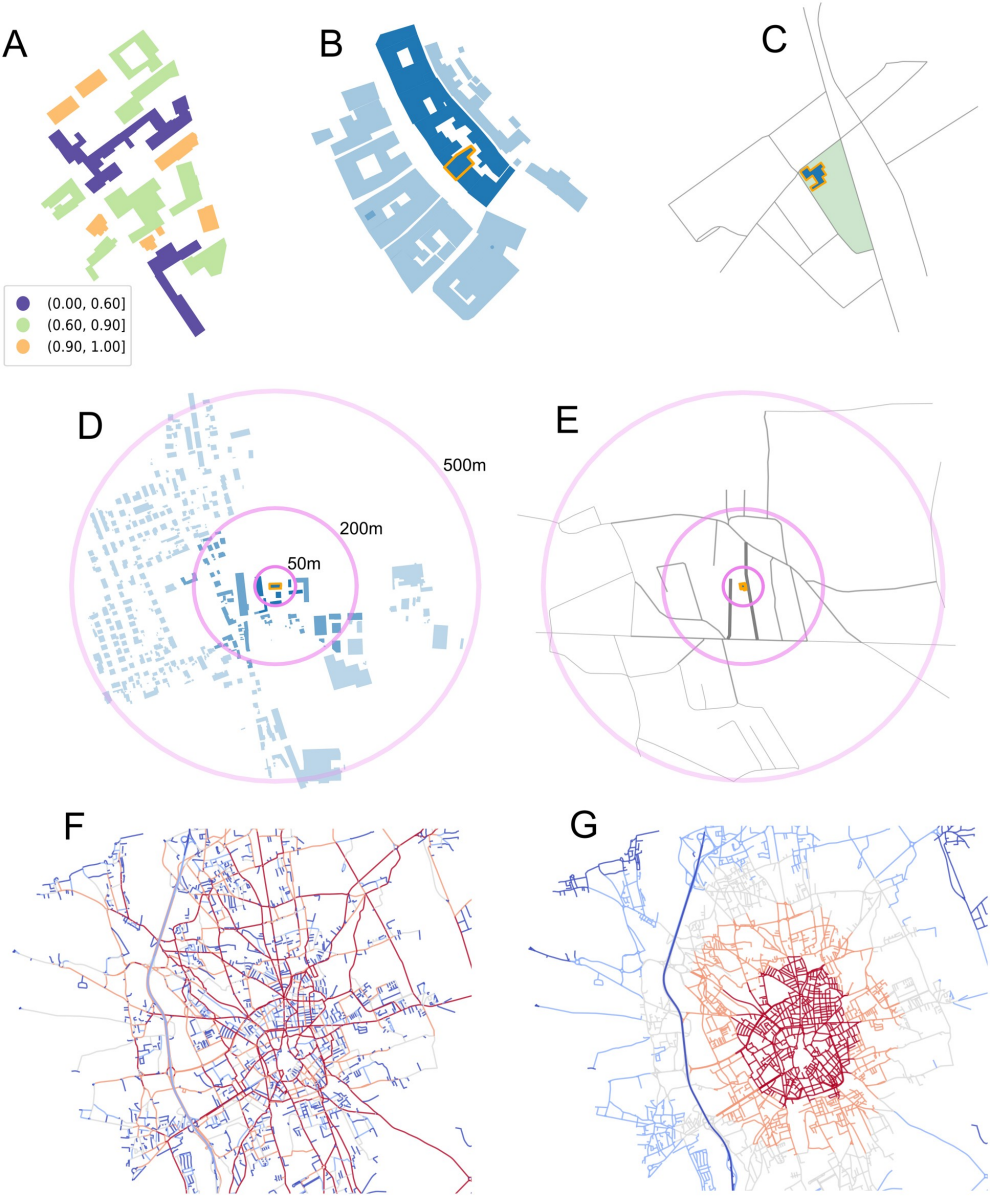
Illustrations of the urban form features used. **(A)** Individual building footprint geometries. The convexity values of buildings’ footprint polygon are displayed in the legend. Convexity ranges between 0 and 1. **(B)** Block of adjacent buildings. The block in which a building of interest is located is depicted in dark blue. **(C)** Street-based block, in green, surrounding a building of interest. **(D)** Buildings within a circular buffer of 50, 200 and 500 m around a building of interest. **(E)** Streets within a circular buffer of 50, 200 and 500 m around a building of interest. **(F)** Betweeness centrality shows main streets and secondary streets. We use as features for example the betweeness centrality of the closest street, or the average within a buffer. **(G)** Closeness centrality shows where streets are converging. Both centrality measures give information on the structure of the city and relative position of a street in the city street network.

From 2D urban morphology data on buildings and street networks, we create 152 features, making use of domain knowledge from urban science, in particular urban morphology and spatial network studies e.g. [25, 26, 43]. Below, we provide rationales for different feature groups and their relation to building height. The features include building footprints, footprints of blocks of adjacent buildings, street segments, street intersections, and street-based blocks, some of which are illustrated in Fig 2A summary table of the features is available in Table 2 (see S2 and S3 Appendices for additional details).

**Table 2:** Summary of urban form features used in this study.

Feature group	Subgroups	Examples	Count
*Buildings*	Building’s own geometry	Footprint area	10
	Building’s own block (if any)	Block elongation	10
	Other buildings within 50, 200 & 500 m	Mean building convexity within 200 m	10 × 3
	Other blocks within 50, 200 & 500 m	Buildings within buffer	8 × 3
*Streets*	Closest street	Betweeness centrality of the closest street	8
	Closest intersection	Distance to closest intersection	1
	Streets fully within 50, 200 & 500 m	Standard deviation street lengths within 200 m	3 × 3
	Streets within 50, 200 & 500 m	Sum street lengths intersecting a 200 m buffer	7 × 3
	Intersections within 50, 200 & 500 m	Count intersections within 50 m	1 × 3
*Street-based blocks*	Building’s street-based block	Street-based block’s corners count	2
	Street-based blocks within 50, 200 & 500 m	Average area street-based block within 500 m	6 × 3
*City level*	Geometry boundaries	Total area city	2
	Buildings	Average building footprint area	3
	Blocks	Count blocks composed of 11 to 20 buildings	6
	Streets	Standard deviation street lengths	3
	Intersections	Total intersections	1
	Street-based blocks	Total street-based blocks	3
Total			**152**

#### Building geometries

The geometries of the building’s footprint itself hold predictive information about its height [24]. In particular the footprint area alone has a strong predictive power [24], and we include another 9 metrics such as the perimeter of the footprint or the footprint convexity (Fig 2A). These additional features help distinguish typologies of buildings based on their form in addition to their footprint size.

It should be noted that buildings are not consistently represented in the different data sources, and even in different regions within OSM. Individual buildings can be represented by several footprint polygons, or several buildings can be combined in one polygon. Due to the large spatial scale of this study, we interpret each polygon as an individual building and accept a possible negative impact on the model performance. In follow-up work, OSM quality assessment methods could be used to reduce these effects [29, 44].

#### Neighborhood

The surroundings of a building contain important information about its height. Including features representing the immediate neighbourhood of a building can also help capture some of the spatial auto-correlation in the data.

We summarize the urban morphology of neighborhoods within circular buffers around the building of interest, see Fig 2D and 2E. We use three scales—50, 200 and 500 meters—in order to capture information about the immediate surroundings of the building, but also the broader neighborhood. Within these buffers, we aggregate information by counting and measuring geometries, and also compute second-order metrics like the average or standard deviation, for example of building footprint areas or street lengths. For these features, we use building footprints, street intersections, street segments and two notions of blocks.

We are interested both in describing the blocks in which a building falls, and the other blocks in the surrounding, again using buffers. The first notion of block is based on buildings immediately adjacent to the estimated building (see Fig 2B). Buildings in the same block tend to be of similar height. Here, within a block, we characterize how similar other building characteristics are (e.g. do all buildings have the same footprint area?) and what the overall features of the block are (is this a long block? is it rectangular or has it a more complex shape?). The second notion is based of an area enclosed by streets [43] (see Fig 2C). Street-based blocks describe well the geometric patterns of the street network in a neighborhood. For example, if the standard deviation in street-based block areas within a buffer is low, and shapes rectangular, in Europe we may be in a residential area with low-rise buildings.

#### Within-city location

Beyond the surroundings of the building, the location within the whole city may also hold predictive value. For example, denser areas in city centers tend to have higher buildings. We use network-theoretic metrics using the streets of the city as edges and the intersections as nodes to determine how central a building is located, see Fig 2F and 2G. We use closeness centrality to measure how close or far the building is from the area of the city where most streets intersect, and betweenness centrality to describe if the building is located close to main roads. To build the features, we compute these metrics for the closest street to the building, and take the average and standard deviations of values within buffers as well.

#### City type

Finally, characteristics of the city as a whole are used to learn inter-city variation. We compute aggregate metrics for each of our feature groups, for example total number of blocks or average footprint area in the city. We also use metrics to describe the shape of the administrative boundary of the city and the total area.

### Prediction models

We compare four prediction models—the median height as the baseline, linear regression, and two tree-based ensemble algorithms, random forest [45] and gradient boosting [46] in the XGBoost implementation [47].

Random forests have already been successfully applied to predict building heights [22]. We additionally used XGBoost because of its computational efficiency. Both these tree-based ensemble methods can handle the interactions between many predictors well, and function with correlated predictors. We compared the results with a simple baseline model that takes the median of the building heights in the training dataset as the prediction for all data points. Finally, to evaluate whether the relationships between heights and our features are linear or non-linear, we also fitted a linear model.

## Experimental design

We explore the abilities of our model to predict building heights across Europe and simulate the conditions of real-world deployment for predicting areas where 3D models are missing in Europe. We conduct three experiments to evaluate the generalization of the model to unseen data from other countries, and to investigate if the performance can be enhanced by access to local data. For details on the experimental procedure see S4 Appendix.

### Training and evaluation

We chose the areas of Berlin and Brandenburg as test sets, which differ in their distributions of medium height and tall buildings. We used the remaining data (five French urban areas, Friuli-Venezia Giulia and the Netherlands) for training and cross-validation. This resulted in around 9 million data points for training and model selection, and around 2.5 million for testing the model.

We use several metrics to evaluate the models’ performance. In the following, we use $h_{err}=\hat{h}-h$ to refer to the prediction error, where *h* is the ground truth height and $\hat{h}$ is the predicted height. For planners and modelers, it is important to have a correct estimate of the number of floors in a building, corresponding to a prediction error *h*_*err*_ smaller than the floor-to-floor height values, which vary across buildings. We use a conservative floor-to-floor height of 2.5 meters that is in the range of legal minimum floor-to-ceiling requirement in our four countries of interest (between 2.2 m and 2.7 m) [48–51].

To assess the overall ability of the models to generalize, we computed three standard set-level metrics: the mean absolute error (MAE), root mean squared error (RMSE) and coefficient of determination (R^2^). We use these metrics for validation and testing. In addition for testing, we computed the overall percentage of buildings with an acceptable prediction error for planning purposes (where *h*_*err*_ < 2.5 m). For the test set, we apply further metrics to understand if we can predict well across building heights *h*. We evaluate the distribution of the errors, and calculate the percentage of buildings where *h*_*err*_ < 2.5 m for different building height ranges where *h* ∈ (2 *m*, 5 *m*], *h* ∈ (5 *m*, 10 *m*], *h* ∈ (10 *m*, 15 *m*], and *h* > 15 *m*. We also differentiate across cities by computing the RMSE, MAE and R^2^ at the city level, and then group by city size. Finally, to evaluate the importance of urban form features, we use their average *gain*, which represents each feature’s contribution for each tree in the model.

We performed a spatial cross-validation by training on folds that are geographically distant, which is expected to improve the generalization of the model [52]. As the dataset for the Netherlands is much larger than the two other areas used for the cross-validation, we split the Netherlands in two folds—thus resulting in four different folds in total (Netherlands 1 and 2, Italy, France).

All four models—median height baseline, linear regression, random forest and XGBoost—are tuned, evaluated, and compared with this cross-validation procedure. For the linear regression, we used the standard scikit-learn implementation [53], included all the features and assumed no interactions between the predictors. For the random forest, we tuned manually a few important hyperparameters, for example the number of trees. For XGBoost, the hyperparameters are tuned through a randomized grid-search with cross-validation [54]. We used 500 combinations of 7 parameters on 4 folds, resulting in 2000 fits. Then, we selected the model based on the best mean absolute error, see S4 Appendix for details.

**Fig 3:**
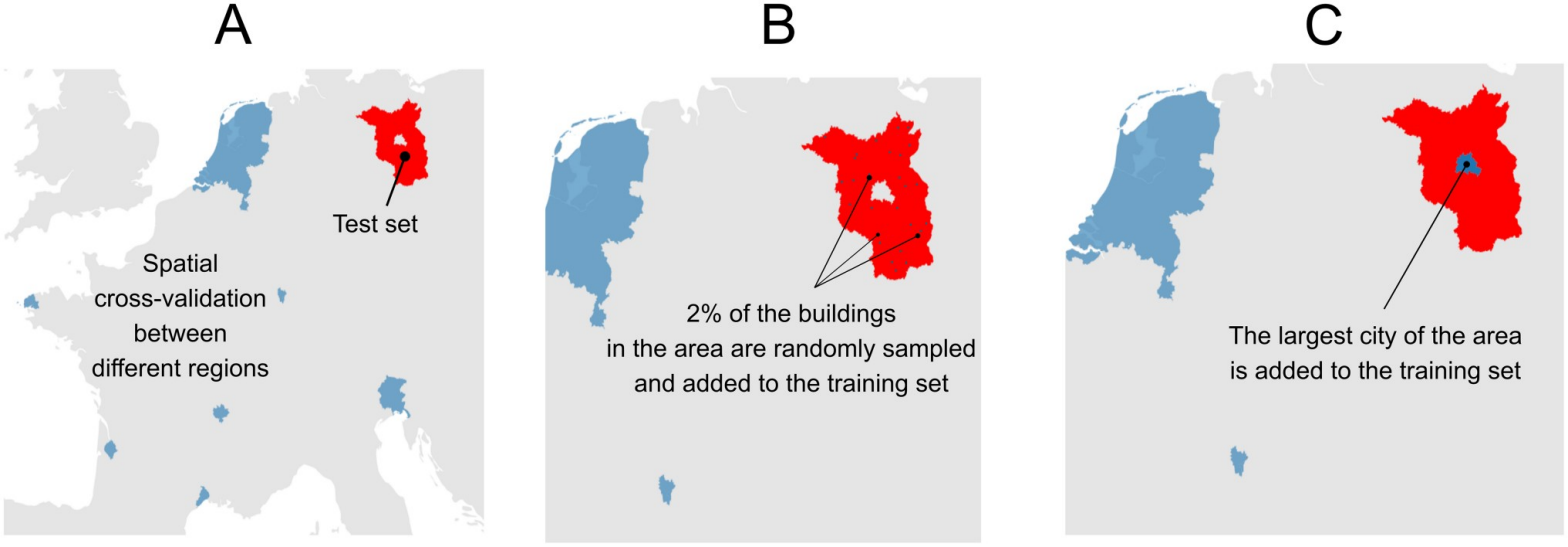
Map of the three experiments on the test set of Brandenburg. **(A)** Experiment 1: No local data are available, and the model is trained only on data from other countries. **(B)** Experiment 2: Scarce local data are available—we add 2% of the test set to the training set, to test the hypothesis that these data provide relevant data for training the model. **(C)** Experiment 3: The main city of the area is available—we add this city to the training. The areas in blue are the training set and the area in red is the test set.

We used the model selected through the cross-validation to conduct the following three experiments on two test sets, Berlin and Brandenburg, see Fig 3 for the experimental setup by the example of Brandenburg. All experiments are evaluated on the same two test sets for Berlin and Brandenburg.

### Implementation

In all experiments, we use the model selected through the cross-validation and trained on the five French urban areas, Friuli-Venezia Giulia and the Netherlands to predict building heights in each of the test areas (Brandenburg and Berlin).

#### Experiment 1: No local data are available

This model setup is our default model with no local data from the test area used for training, see Fig 3A for the example of the test on Brandenburg. Data availability varies widely between countries with some regions having no local data at all. We first test how well the model generalizes to our test areas under these conditions. We aim for the model to learn relevant attributes across contexts, as opposed to overfitting to one.

#### Experiment 2: Scarce local data are available

In this experiment, we add a random sample of 2% of the buildings from each of the test areas and add these samples to the training set respectively, see Fig 3B. In reality, most areas have at least a few datapoints of building heights available from OSM, which can be as high as 90% like in the case of Friuli-Venezia Giulia. More often, however, less than a few percents of the buildings have height data in OSM. For example, in Berlin only ∼2% of OSM buildings have heights, despite Berlin’s active mapper community (see analysis in S9 Appendix). With this experiment, we explore whether such limited local data can help improve the accuracy of predictions.

We randomly sample 2% of the buildings in Brandenburg and Berlin, respectively, and add it to the training set. This represents around 9,000 buildings for Berlin and around 43,000 for Brandenburg—but only a tiny fraction of the training data (0.09% and 0.6% respectively). To mitigate potential sampling effect, we repeat this experiment five times and report average results. By randomly sampling in official data, we make the implicit assumption that the quality of the available data would be high, while it may not be the case with heights reported in OSM. Here, we also use a sample representative of buildings heights distribution—although not necessarily of buildings types. In reality, there may be biases towards certain heights and types of buildings in the OSM sample.

#### Experiment 3: Main cities of the area are available

In this experiment, we add the city of Berlin to the training set, see Fig 3C, and test only on Brandenburg. Large cities are more likely to have the means to acquire a 3D model, and in several countries official height data for only few large cities are available. With this experiment, we test if local data from large cities can help to improve the prediction performance for rural areas in these countries. The hypothesis underlying this experiment is that these data on large cities contain country-specific characteristics that helps the prediction task.

## Results

To better understand the potential of our model to predict in new areas in Europe, we are interested in performances of the model along several dimensions. First, which of the chosen models performs best on the validation data and could be used? Second, can such a model generalize to unseen data in another country? Third, does the model perform better or worse in certain contexts, in particular with respect to different building heights and city types. Fourth, can the scarce data available in practise improve the learning? Fifth, which urban form information is most predictive of building heights?

### Model selection

With the cross-validation procedure, we evaluated the four models on geographically distant folds (Netherlands 1 and 2, Italy, France) to select the best performing model. The tree-based methods performed better than the linear regression and the baseline (Table 3). The XGBoost model tuned with grid search had the lowest average MAE of 1.47 m averaged over all four areas. Based on this result, we used XGBoost for conducting our experiments on the test sets. See S4 Appendix for the details on the model parameters chosen.

**Table 3:** Model comparison results.

	Italy			France			Netherlands 1			Netherlands 2			Average CV	
Model	MAE	RMSE	R^2^	MAE	RMSE	R^2^	MAE	RMSE	R^2^	MAE	RMSE	R^2^	MAE	RMSE
*Median h baseline*	2.35	3.29	–	3.17	5.14	–	2.11	2.86	–	1.99	2.69	–	2.41	3.50
*Linear regression*	2.10	2.93	0.20	2.69	4.43	0.22	1.73	2.36	0.30	1.60	2.24	0.31	2.03	2.99
*Random forest*	1.78	2.60	0.38	2.42	4.12	0.33	0.96	1.65	0.66	0.93	1.63	0.64	1.52	2.5
*XGBoost (tuned)*	1.65	2.60	0.38	2.38	4.06	0.36	0.94	1.68	0.66	0.91	1.61	0.64	1.47	2.49

Summary of the mean absolute error (MAE), the root mean squared error (RMSE) in meters and the coefficient of determination (R^2^) of the different models. The baseline model makes a constant prediction based on the median of the building heights in the training data. For each region, values correspond to predictions in the region after training on the *n* − 1 other regions.

The random forest achieved similar results to XGBoost for the two Dutch regions and France, but performed worse for Italy. Both tree-based ensemble methods significantly outperform the other models. The baseline has average prediction errors in the range of several meters, which corresponds to a high rate of mispredicting the number of floors. The linear model achieved a MAE of around two meters on average.

These results also need to be put in perspective by comparing the computational efficiency of the algorithms: training the different models on the entire data set using a single CPU took minutes for the linear model, hours for XGBoost and days for the random forest.

The two folds in the Netherlands provide the closest comparison to the benchmark study of Biljecki et al. [22], which uses two Dutch cities as their case study. Our best model here achieved a MAE of 0.91 m on one fold. This is comparable to the performance of their best model (0.90 m), where they trained a random forest on 10% random sample of Rotterdam and predicted on the other 90%. For predicting an unseen city, Biljecki et al. achieved a MAE of ∼1.1 m. We used a much larger training set but did not include the number of floors of the buildings as a feature.

### Cross-country generalization is possible

**Fig 4:**
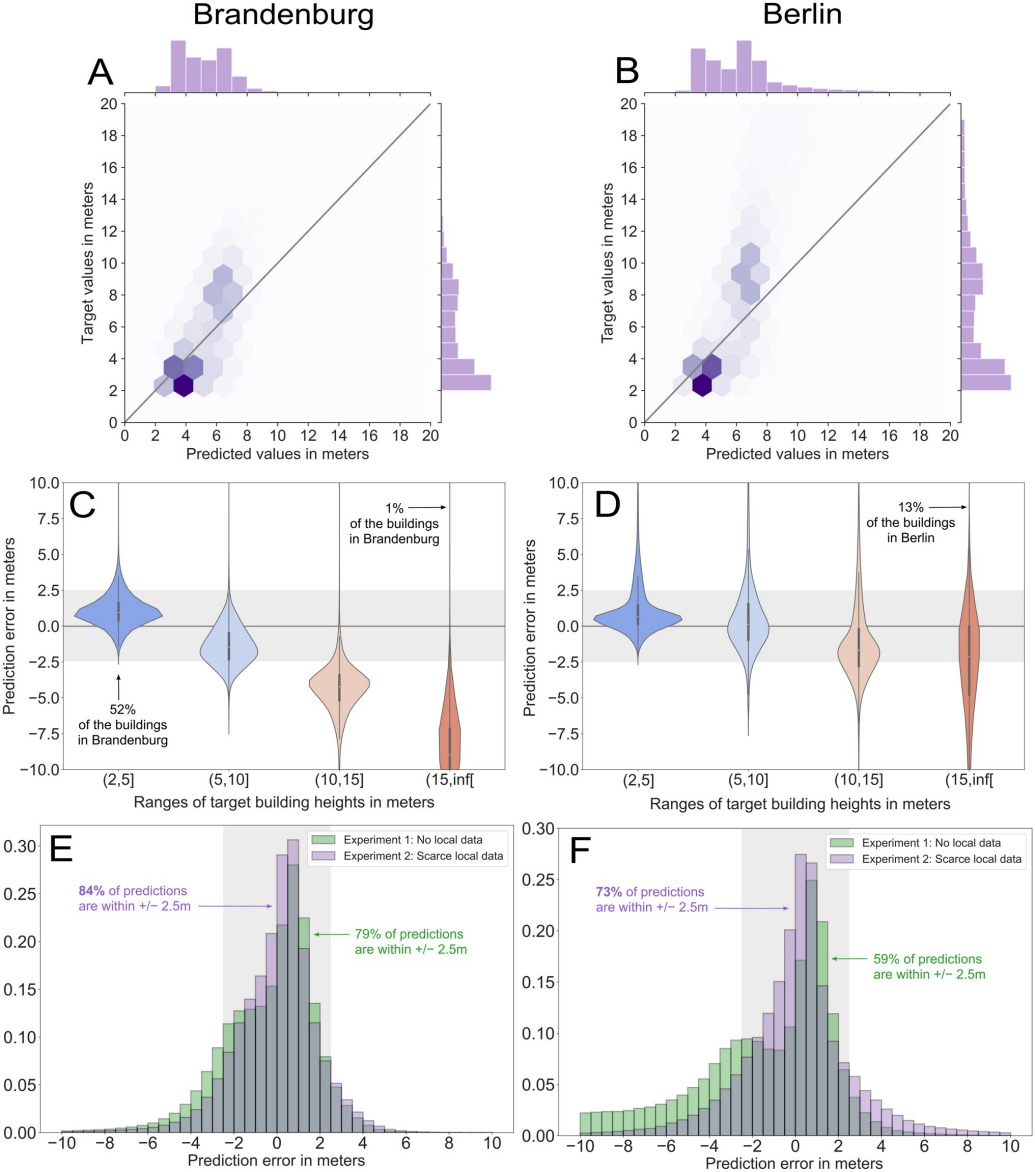
Results of the predictions for the test regions, Brandenburg (*left*) and Berlin (*right*). **(A–B)** Joint plot of predicted values over target values for *Experiment 1* (No local data), both in meters, with the marginal distributions as barplots. The intensity of the bins’ color represent the density of data points in the bin. On the thick diagonal grey line, points are perfectly predicted. Points above the line are under-predicted and those below the line are over-predicted. **(C–D)** Error distribution of different target height ranges, for *Experiment 1* in Brandenburg and for *Experiment 2* (2% local sample) in Berlin. The shaded areas represents an error range of +/− 2.5 meters, which roughly corresponds to the height of one floor. **(E–F)** Error distributions for *Experiments 1* and *2*. The shaded areas represents an error range of +/− 2.5 meters.

Without local data used for training in *Experiment 1*, we obtained a MAE of 1.72 m on the test set for Brandenburg and 2.98 m for Berlin (see Table 4). The result for Brandenburg shows that it is possible to predict building heights in a large area with an average error well below the typical floor height (about 2.5 m), without having any training data from that area, not even that country, available. In Berlin, the MAE is larger, likely due to the larger amount of high and unusual buildings. In Brandenburg, 79% of the prediction errors *h_err_* are below 2.5 m compared to 62% in Berlin (Fig 4E and 4F). Also for both test areas, we see a clear improvement of our model over the baseline based on the constant prediction using the median.

**Table 4:** Results of the experiments of the test sets.

	Brandenburg (90%)			Berlin (90%)		
Experiments	MAE	RMSE	R^2^	MAE	RMSE	R^2^
*Median h baseline*	2.56	3.57	–	4.87	6.84	–
*Exp. 1: No local data*	1.72	2.72	0.41	3.39	5.32	0.41
*Exp. 2: Adding a 2%-sample*	1.47^+^	2.37^+^	0.53^+^	2.06^+^	3.50^+^	0.72^+^
*Exp. 3: Adding a large city*	1.72	2.66	0.41	–	–	–

Summary of the mean absolute error (MAE), the root mean squared error (RMSE) in meters and coefficient of determination (R^2^) of the different experiments on the test sets, using XGBoost with hyperparameters obtained through cross-validation on the training set. In order to have an identical test set across experiments, 90% of the data in Brandenburg and Berlin are used, after removing five times a 2% sample for *Experiment 2*. The baseline model makes its predictions based on the median of the building height distribution (4.68 meters for Brandenburg and 6.69 meters for Berlin).

^+^Average over five configurations with different samples of 2% of the test set.

The results of the cross-validation suggest that the test results are robust and the model can generalize across Europe, as a MAE below 2.5 m was achieved in all four cases. Errors are larger for Italy and France, which are smaller, more specific datasets (mostly rural and mostly urban, respectively). The two folds in the Netherlands had very similar distributions and millions of data points for training. This may explain the good performance on these data sets.

The coefficient of determination was 0.41 for Berlin and Brandenburg, and ranged between 0.38 and 0.66 for the cross-validation. This shows that the model was able to reproduce a good proportion of the variance in building heights across the different countries.

### Low buildings were predicted more accurately than high ones

The model predicts most of the test set’s building heights well, but the accuracy decreases for buildings taller than 10 meters in *Experiment 1*. Fig 4A–4C illustrate the performance on the test region of Brandenburg and Berlin by height for *Experiment 1*.

The model has a tendency to strongly underpredict higher buildings, and slightly overpredict the smallest buildings in both Brandenburg and Berlin (Fig 4A–4C). In particular, buildings with *h* ∈ (2 *m*, 5 *m*] were predicted best: 90% and 89% of those buildings had an *h*_err_ < 2.5 *m* in Berlin and Brandenburg respectively. The error increased with building height, and for most of the buildings with *h* > 10 *m*, the predictor error was not within a 2.5 m range. However, it is important to note that in Brandenburg, as in most regions, buildings under 10 meters account for 91% of the distribution. In Berlin, a more urbanized area with higher buildings, this percentage drops to 72%.

Building heights in smaller cities in Brandenburg were generally predicted more accurately compared to those in larger cities. In *Experiment 1*, the MAE was 1.63 m for cities up to 5,000 buildings, 1.72 m for cities with 5,000 to 20,000 buildings and 1.89 m for cities above 20,000. The probable reason for this is that larger cities generally have a higher proportion of tall buildings for which predictions are less accurate.

### Additional local data can improve the accuracy

In *Experiment 2*, we added 2% of local data to the training set data which resulted in noticeable accuracy gains compared to *Experiment 1* for both test sets. In contrast, *Experiment 3* where we added Berlin to the training set for predicting Brandenburg did not noticeably improve the results.

**Fig 5:**
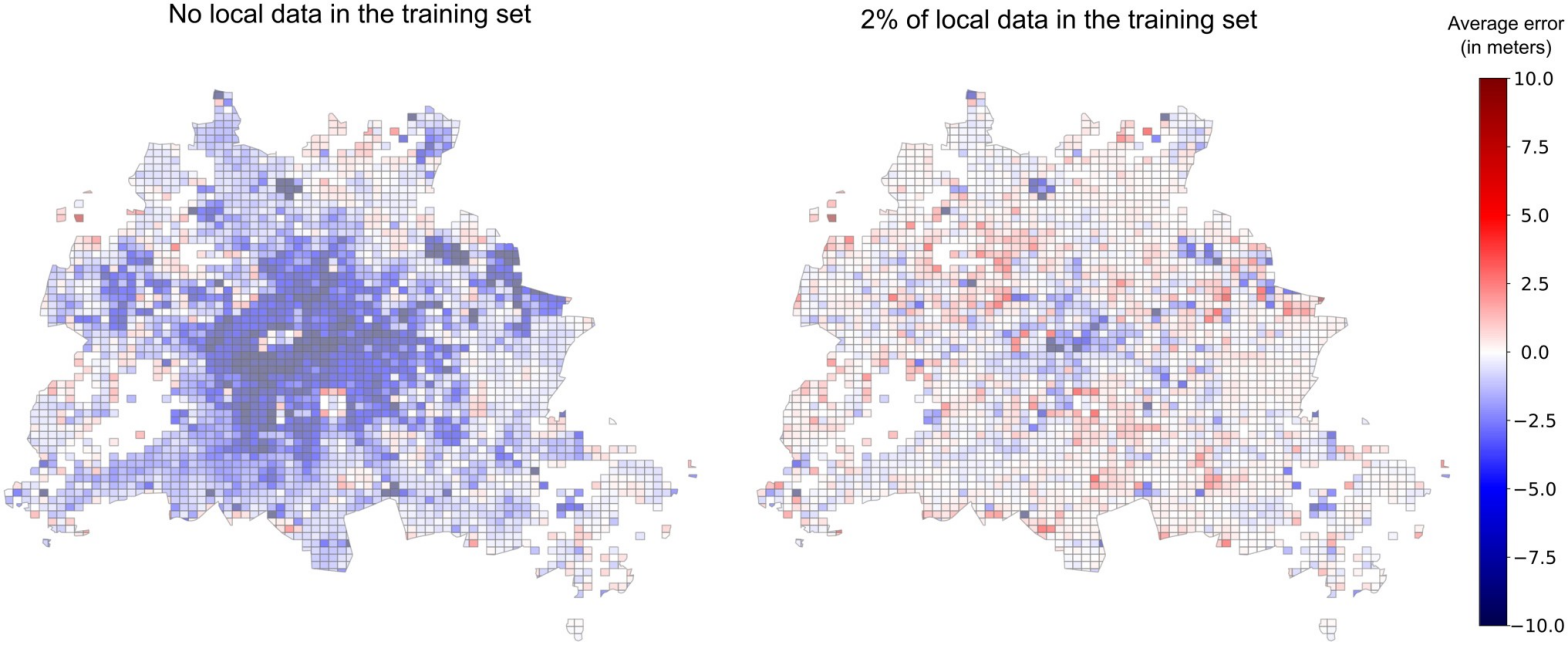
Prediction errors in Berlin for Experiments 1 (no local data) and Experiment 2 (2% of local data). The errors (in meters) are aggregated on a grid for better readability, and depicted by a color gradient. The presence of local data in Experiment 2 starkly reduces the errors and the occurrences of under-prediction, especially in the center of Berlin.

The test MAE improved by 1.03 m and 0.25 m for Berlin and Brandenburg, respectively, with *Experiment 2*. These improvements represent a substantial gain over *Experiment 1*, see Fig 5 for a visualization for Berlin. The total percentages of errors *h_err_* below 2.5 m improved from 79 to 84% in Brandenburg, and 59 to 73% in Berlin, see Fig 4E and 4F.

The model could also reproduce an additional 31 and 12% of the variation in *h* for Berlin and Brandenburg (see Table 4). In particular for Berlin, adding scarce local data enabled to reduce cases of undeprediction for high buildings, as shown in Figs 4E and 4F and 5. For tall buildings above 10 m, the proportion of buildings with prediction errors below 2.5 m increased remarkably from a few percents to more than 50%, see Fig 4D. (See S2 and S3 Figs for more comprehensive figures to compare the errors distributions.) One potential explanation may be that this additional training helped the model recognize a morphology typical to Berlin, for example the residential areas with townhouses from the early 20^th^ century (’Altbau’) that have 5-6 floors and similar heights. The performance increase is particularly astonishing considering the minuscule amount of new data points that were added to the training dataset in *Experiment 2* compared to *Experiment 1*.

Against expectations, adding the city of Berlin to the training set yielded no accuracy improvement over the performance on Brandenburg in *Experiment 1*. The MAE is 1.72 m and the R^2^ is 0.41 in both cases. Metrics by height range or at the city-level also had very similar results for the two experiments. This could be explained by the fact that Berlin as the historical capital has different types of street patterns. Also, the townhouses that are so typical for Berlin are not as common even in large cities in Brandenburg, despite the geographical vicinity.

### The diversity of urban form features helps prediction

All feature groups—buildings, blocks, streets, street-based blocks, city-level—were found to increase the prediction performance. The feature groups with the highest *gain* across folds and experiments are buildings and city block features (see Table 5).

**Table 5:** Feature importance.

Features	Count	Sum Importance	Avg Importance	Highest individual feature	Highest gain
*Per group*					
Buildings	40	41%	10‰	Footprint area	11%
Blocks	40	40%	10‰	Block perimeter	5%
Streets/intersections	42	11%	2‰	Distance to closest road	1.5%
Street-based blocks	21	4%	2‰	Count blocks intersecting a 500m-buffer	1%
*Per scale*					
Footprint & closest	31	51%	16‰	Footprint area	11%
50 m	33	8%	2‰	Total footprint area	1%
200 m	33	10%	3‰	Average block area	1%
500 m	33	22%	7‰	Average building count in block	5%
City-level	16	8%	5‰	Average building footprint area in blocks	1%

The feature importance scores are reported for the best experiment, Exp. 2. The importance metric used is the average gain across all splits the feature is used in. We report features aggregated by feature groups and scales. Because the clusters are imbalanced, we also report average importance per cluster.

Among individual features, the building’s footprint area is always by far the most important, and other building-level features like the perimeter or the length of shared walls with other buildings often have high importance. When comparing the importance of individual features, the most important group are the block features, see S1 Table. Those include the total perimeter of the block of a building, the average and standard deviation of blocks’ footprints within 500 m and also the average footprint area of buildings that in a block within the whole city.

We also compared different scales that features apply to and find that all scales carry importance. The building’s own geometry, and the closest street and intersection account for half of the *gain* (see Table 5). The urban context within a distance of 500 m is more predictive than within 200 m and 50 m. The features describing neighborhood seem in general more predictive than those describing the location of the building in the global street network of the city. The aggregate city-level features also proved important.

The predictive power of those features that describe the neighborhoods of buildings (as measured by the sum of the gain values) indicates that they might capture some of the spatial auto-correlation of building heights. We analyzed the spatial auto-correlation of the predictions and residuals, for models with and without these features in S8 Appendix. Across all experiments, the model with the full set of features is better able to reproduce the spatial auto-correlation of the target building heights, compared to the experiments where those features describing the neighborhood of the building of interest were removed. In most cases, neighborhood features also reduced the spatial auto-correlation in the residuals substantially.

## Discussion

The main objective of this study was to examine to what extent it is possible to improve the availability of up-to-date height data in Europe at low cost and without recourse to proprietary data. The collection of primary data is time- and cost-intensive, and therefore often carried out with limited temporal and spatial resolution. Our results show that such an approach is feasible, but they also highlight remaining challenges with predicting high buildings. The accuracy of our model achieved so far may already be sufficient to inform regional policies and studies, for example, estimating the energy demand of a large building stock. Some applications require more precise results, such as neighborhood planning, where building-level exact measurements are more applicable. The promising results of combining citizen-generated data with open government data to deliver policy-relevant information should encourage the public sector to increase its support for open data strategies.

### Towards an open European infrastructure database

Our method can serve as a step towards a continuously updated, open and comprehensive building stock model at the resolution of individual buildings for Europe. For the purpose of testing our proof-of-concept, we have limited ourselves to predicting two areas (Brandenburg and Berlin) where an open 3D building model exists. We validated the approach by assessing how the model generalizes to new areas with data from four different countries. Based on the analyses, we think that the prediction can be extended to regions and countries for which only OSM building footprints are available. This bears the potential to create a database of building heights estimates that covers the whole of Europe.

This study of predicting building heights gives reasons to assume that similar approaches have the potential to use urban form data for predicting missing urban data more generally. There are other infrastructure elements in cities where urban morphology influences how these elements evolve. In the case of buildings, for example, building use (e.g. residential or commercial) or building age are likely to be quantitatively related to urban form. Also, usage rates of urban infrastructure, e.g. transport demand, could be predicted with such an approach, since they depend strongly on urban form [3, 14].

It is difficult to estimate how well the model applies outside Europe. Firstly, the structure of urban areas can be very different from those found in Europe, as well as the distribution and meaning of some features. Secondly, the availability of training data and the completeness of building footprints is still very limited in many areas. New methods, such as estimating building footprints based on remote sensing, can potentially help here. This is of interest for future analysis.

### Opportunities for the public sector

Our study shows the high potential value that VGI can have for evidence-based, open governance. Contributions from individual citizens, who add information about building heights in their neighborhood to the OSM database, can help improve data-driven models estimating building heights. Our results have shown that even scarce but localized information significantly improve the predictive power of the model. In turn, our methodology can help improve the overall availability of attributes that are scarce in OSM and contributes to the literature aiming to ‘fill the gaps’ in OSM e.g. [55]. An alternative OSM attribute of high relevance to a model like ours is the number of floors of a building [22], which is easier to map because directly observable in most cases.

Our approach, if scaled-up, offers cities the opportunity to obtain valuable data on their infrastructure at low cost. Democratic decision-making processes for the common good depend on transparency and participation. As a result, there is an obligation not to rely exclusively on private, profit-oriented companies to acquire and govern the data required for essential functions of public governance. This creates a strong incentive for the public sector to invest more in open data and VGI. After all, predictions based on supervised learning approaches are not possible without high-quality training data, and open government data are a major source of such reliable data. Our findings also suggest that data from different contexts are needed to achieve good performance and underline the relevance for all cities to continue to engage in open data strategies.

### Improving and scaling the approach

A main caveat of this study is the insufficient prediction accuracy for high buildings, which has methodological implications. Reasons include i) data have a lower bound (0 m) but no higher bound, allowing for higher variance in high building estimates; ii) training data has a much larger fraction of low buildings than high buildings; and iii) features of higher buildings may be more diverse than low buildings (a hypothesis to be tested).

We ran several sensitivity analyses to address the performance of the model vis-a-vis some of these concerns. First, to address ii), we balanced the distribution between high and low buildings by uniformly sampling from height bins (see S5 Appendix). This experiment did not improve the prediction accuracy of high buildings for Brandenburg. The result may be specific to Brandenburg and requires further investigation. Second, we trained the model on data constrained to small- and medium-sized cities to analyze if those models show better performance when tested on rural regions (see S6 Appendix). We found the performance change to be small (a few centimeters) and the original model in some cases even outperformed those models. Third, we removed high buildings beyond 20, 30 and 40 meters, to test if a model optimized on smaller buildings would bring a performance gain (see S7 Appendix). We found this set-up to only marginally improve the performance (the MAE improved of −2 to −6 cm).

We plan to upscale this proof-of-concept with more data both for training and testing, with a particular attention on adding new geospatial contexts that could form a broader and more representative dataset. We expect that this would improve the learning, while it will enable to test our hypotheses at a larger scale. In the future, we also aim to predict to areas without an available 3D model using OSM data. To further analyse the abilities of the model to generalize in follow-up studies, the ‘area of applicability’ approach [56] might be an interesting starting point. Prediction uncertainty could be investigated further relying on recent developed methods for the XGBoost algorithm [57].

Finally, there are opportunities to use other algorithms and training procedures, including using raster images of urban tissue geometries directly with computer vision algorithms. Also, spatial auto-correlation could be leveraged beyond the set of features we have included so far (see S8 Appendix for a discussion of the spatial auto-correlation in our data and model output). The emerging sub-field of spatial machine learning provides new approaches that are more tailored to spatial data than the methods used here [58–60].

## Conclusion

The vertical dimension of urban infrastructure is key to improving our understanding of cities and design policies to adapt cities to sustainable futures. It is important that such building height data are openly available at large scale. In this proof-of-concept, we showed that machine learning models can predict building heights based on data from public sources as well as volunteered geographic information. Our approach uses features based only on geometrical data on urban form in 2D and domain knowledge from urban studies. Since these data are available with good coverage in Europe, our approach constitutes a step towards filling data gaps in the entire region. The model’s predictions generalize well across countries, but have larger prediction errors for tall buildings. We also find that if limited local ground-truth data is used for training, prediction performance improves substantially. These results stress the value of individual contributions from OpenStreetMap mappers for the public sector.

## Supporting information

S1 Appendix Data preprocessing. In this appendix, we summarize how the data preprocessing has been carried out. The whole workflow for preprocessing, feature engineering and machine learning is written in Python and executed on the high performance computing cluster of the Potsdam Institute for Climate Impact Research.
(PDF)

S2 Appendix Feature engineering. In this appendix, we explain in detail how each feature group has been implemented.
(PDF)

S3 Appendix Complete feature list. This appendix contains all individual features with their full name, unit, variable name, and, when relevant, their definition and their source.
(PDF)

S4 Appendix Machine learning experimental procedure. This appendix describes the details of the machine learning experimental procedure. The algorithms were trained on 32 CPU cores and 128GB of RAM, using the high performance computing cluster of the Potsdam Institute for Climate Impact Research.
(PDF)

S5 Appendix Experiment with artificially balanced training set. (PDF)

S6 Appendix Sensitivity analysis: Specialized rural model. (PDF)

S7 Appendix Sensitivity analysis: Discarding high outliers. (PDF)

S8 Appendix Spatial auto-correlation analysis. (PDF)

S9 Appendix Comparison building heights between OSM and the 3D model. (PDF)

S1 Fig Results of the cross-validation, by fold. *Left*: for each fold, joint plot of predicted values over target values, both in meters. The intensity of the color of the bins represent the density of data points in the bin. On the thick diagonal grey line, points are perfectly predicted, and the light greys lines represent a + /− 2 meters error interval. *Right*: For each fold, error distribution of different target height ranges.
(TIF)

S2 Fig Jointplot of prediction errors for Experiment 1 (*top*) and Experiment 2 (*bottom*) for Brandenburg (*left*) and Berlin (*right*).
(TIF)

S3 Fig Violin plot of prediction errors by height ranges for Experiment 1 (*top*) and Experiment 2 (*bottom*) for Brandenburg (*left*) and Berlin (*right*).
(TIF)

S1 Table Feature importance test set. (PDF)

S2 Table Results of a specialized rural model for Brandenburg. (PDF)

S3 Table Results of removing high outliers from the datasets, for Brandenburg. The table reports the mean absolute error in meters on the test set for each experiment and threshold. Thresholds correspond to the maximum height of buildings included in the training and test sets. We report results for two set-ups: in the first one (diagonal), we remove buildings above a given height in both the training and the test set; in the second, we remove the buildings only from the training set (vertical column with ‘none’ for Test). The results of this table should be compared vertically for a given test set.
(PDF)

S4 Table Moran’s I of the model’s outputs and residuals in various settings for Berlin and Brandenburg. We report a set-level measure of spatial auto-correlation, the global Moran’s I, which is computed in Brandenburg and Berlin for the two experiments, with and without features describing the surroundings of a building. The Moran’s I is computed on both the residuals and the output of the model. Values for the target heights are reported for comparison.
(PDF)

S1 Code Scripts. (PDF)
