# Supplementary material for: Learning from urban form to predict building heights
Source: PLoS One. 2020 Dec 9;15(12):e0242010. doi: 10.1371/journal.pone.0242010 (PMC7725312; doi:10.1371/journal.pone.0242010)
Supplement: S1 Appendix — In this appendix, we summarize how the data preprocessing has been carried out. The whole workflow for preprocessing, feature engineering and machine learning is written in Python and executed on the high performance computing cluster of the Potsdam Institute for Climate Impact Research. (PDF) [file pone.0242010.s001.pdf]

S1 Appendix. Data preprocessing. In this appendix, we summarize how the data preprocessing has been carried out. The whole workflow for preprocessing, feature engineering and machine learning is written in Python and executed on the high performance computing cluster of the Potsdam Institute for Climate Impact Research.

1. *Processing 3D models.* The aim of this step was to save as tabular data for each building a single footprint geometry, height and id. The 3D models used had diverse formats: CityGML (Berlin, Brandenburg, Lyon), tabular (Friuli-Venezia Giulia, the Netherlands), OBJ (Strasbourg, Brest), 3DS (Bordeaux) and DXF (Montpellier).

   Within CityGML models, the height was sometimes available as an attribute. In this case, we used this value. However, in particular for Berlin, there were sometimes missing values for the attribute. Then, we computed the difference between the highest and lowest point of the building. In order to be at least locally consistent, we used this second method for all building heights in Berlin. We also retrieved the id and footprint polygons from the 3D models.

   For height data coming from OpenStreetMap (Friuli-Venezia Giulia), we first surveyed where the `height` attribute was populated using `Osmium` on a local version of the OpenStreetMap planet file. We then used the library `osmnx` [1] to extract the data at the level of cities. The cities we used had a ratio of 'buildings with heights over total OSM buildings in the city' above 90%. We also retrieved the OSM id and footprint polygon.

   For the height data of the Netherlands, we used the 3DBAG from TU Delft. In these tabular data, building heights were available at different percentiles of the point cloud. We chose to compute the height by substracting ground values from roof 75 percentile, as validation work from the data curators indicates this was the closest value to actual heights.

   The data preprocessing for the 5 French cities was carried out separately. The only city where explicit footprint and height information was given for each building was Lyon. For the sake of consistency, we have chosen to use the same three-step pipeline to compute footprint and height for all 5 cities. The first step consisted in computing the difference between 3D geometries representing facades and roof slopes. When this information was not explicitly available in the source, the inclination of the surface was computed through its normal vector: surfaces with an inclination between 0° and 80° were considered as roof slopes and those between 80° and 90° (i.e. close to vertical), facades. In the second step, a spatial join was performed between facade and roof 3D geometries and 2D footprint from OpenStreetMap. The matching between geometries was not always possible and 16.6% of buildings were lost in the process. Finally, the last step consisted in computing a building's height given its OpenStreetMap footprint and the 3D facade and roof geometries associated to it. We computed three different height levels : the lowest point of all the facades, the lowest point of the highest roof and the highest point of the highest roof. The building height was then computed as the difference between the middle of the highest roof (average between its highest and lowest points) and the bottom of the lowest facade.

2. *Matching buildings with administrative boundaries.* In order to have the most consistent definition of administrative boundaries, we used the Database of Global Administrative Areas (GDAM). Note, however that the granularity of the

administrative unit varies across countries (e.g. in the Netherlands over the last decades, villages have been progressively aggregated into larger areas), which could have an impact on several features. We joined spatially administrative boundaries and buildings for each GDAM city in a region. We saved buildings and boundaries in individual files for each cities.

3. *Adding buffer zones.* Because we wanted to compute features about the surroundings of buildings, we needed to add buffers on the outskirts of a city. If not, we would have biased values at the borders of each city. We added a 500 m buffer for the buildings layer, and because streets can be much longer, we added a 2 km buffer for them. We saved the extended boundaries in the individual city boundary file.

4. *Parsing street networks.* Street networks used in this study are all from OpenStreetMap. We used `osmnx` [1] to retrieve streets for each city using the 2 km buffer. We kept only one way when there were two-way streets. We also kept only drivable streets, following the assumption that the quality of these data would be more consistent across regions than for more specific street types, and as the loss of information from these streets seemed acceptable, especially from a global network perspective. We then used the library `momepy` [2] to compute network metrics. Finally, we converted the nodes of the networks into (street intersection) points, and the edges into (street) lines, and saved each as separate files for each city, including as additional attributes the computed metrics and their ids.

5. *Generating street-based blocks.* When trying to capture morphology metrics on street-based blocks, we found that it seemed simpler to view them as polygons rather than lines or networks. One can then compute area and shape metrics in a straightforward way. Therefore, we converted street lines into polygons, and saved these 'street-based blocks' into individual files for each city.

6. *Adjusting city extent.* Because the functions for feature engineering were computationally intensive and required much RAM for the large cities, we decided to cut cities above 50,000 buildings into parts to parallelize computations. This is done by creating a grid within the administrative boundary polygon, adding a buffer to each part, and joining it to the building geometries. Each part was then saved as an individual file.

References

1. Boeing G. OSMnx: New methods for acquiring, constructing, analyzing, and visualizing complex street networks. Computers, Environment and Urban Systems. 2017;65:126–139.

2. Fleischmann M. MOMEPY: Urban morphology measuring toolkit. Journal of Open Source Software. 2019;4(43):1807.
