# Supplementary material for: Learning from urban form to predict building heights
Source: PLoS One. 2020 Dec 9;15(12):e0242010. doi: 10.1371/journal.pone.0242010 (PMC7725312; doi:10.1371/journal.pone.0242010)
Supplement: S2 Appendix — In this appendix, we explain in detail how each feature group has been implemented. (PDF) [file pone.0242010.s002.pdf]

S2 Appendix. Feature engineering. In this appendix, we explain in detail how each feature group has been implemented.

1. *Building features.* First, we generated a set of ten features at the building level that characterize the geometry of the footprint. Two features (`CountTouches` and `SharedWallLength`) also link the building footprint with that of other potential buildings directly touching it.

   Second, we generated a set of ten features that characterize a potential block, defined as a series of adjacent buildings. Blocks are retrieved by a function that recursively searches next adjacent buildings. These features describe block-level characteristics by the sum of a metric over all buildings in the block, but also the average and standard deviation values between individual building components, which describe the diversity within a block.

   Third, we generated a set of three times ten features – i.e. thirty features – to describe the building footprints surrounding the building of interest within circular buffers of 50, 200 and 500 m around the centroid of the building. For this, we performed spatial joins between the three buffer geometries and building footprints to retrieve the buildings that are fully contained or intersect the buffered area. Then, we computed total metrics for the buffered area, as well as average and standard deviation values between individual buildings to describe the diversity within the area.

   Fourth, we generated a set of three times eight height features – i.e. twenty four features – to describe the blocks surrounding a building of interest within circular buffers of 50, 200 and 500 m. For the retrieval and the metrics calculations, we followed the same approach and rational as for the previous set of features.

2. *Street features.* As for building features, we used several notions of distance for street features.

   First, we retrieved the closest street and intersection from a building. We used these distances as features, and also added metrics characterizing the street and the intersection, including their own properties like length, but also network-level features. This generated a set of eight features. For network features, we used the option in `momepy` to give metrics to edges by doing the mean of the value computed at the node level, e.g. betweeness centrality. We chose this approach as it seemed more representative for a building to be associated with a street in the cases when the closest intersection is far away or when there are many intersections nearby. We also computed features using both buildings and streets, for example the street width (see `momepy` documentation).

   Second, we generated a set of three times eleven features – i.e. thirty three features – to describe streets and intersections surrounding the building of interest within and intersecting a circular buffer of 50, 200 and 500 m around the centroid of the building. These features include network metrics and geometry metrics, and we also used counts, sums, averages and standard deviations as second-order metrics. A particularity of streets compared to buildings is that they can be very long. For this reason, we have not only features where the street has to be fully within the buffer to be counted, but also features where the street only has to intersect the buffer. Thus, we are not ignoring possible relevant streets, while also describing the network strictly within a distance.

3. *Street-based block features.* For street-based blocks, we first generated a set of two features that describe the block in which the building is located. These features describe the area and shape of the block using the polygon geometry.

   Second, we generated a set of three times six features – i.e. eighteen features – to describe street-based blocks surrounding the building of interest within and intersecting a circular buffer of 50, 200 and 500 m around the centroid of the building. As these blocks are based on streets, and therefore can be very large, we also added features where only the intersection with the relevant buffer is needed for the block to be included. These features use geometry metrics, and we also computed counts, sums, averages and standard deviations as second-order metrics.

4. *City-level features.* Finally, for city-level features, we first described the administrative boundary using the GDAM polygon, generating two features for the total area and shape of the city. A caveat is that city boundaries differ across countries in term of granularity, which will affect the relevance of the features.

   Second, we summed for the whole city relevant aspects of all feature groups, generating three features for buildings, five features for blocks, three for streets and three for street-based blocks. Here as well, we used counts, sums, averages and standard deviations.
