# Supplementary material for: Learning from urban form to predict building heights
Source: PLoS One. 2020 Dec 9;15(12):e0242010. doi: 10.1371/journal.pone.0242010 (PMC7725312; doi:10.1371/journal.pone.0242010)
Supplement: S3 Appendix — This appendix contains all individual features with their full name, unit, variable name, and, when relevant, their definition and their source. (PDF) [file pone.0242010.s003.pdf]

S3 Appendix. Complete feature list. This appendix contains all individual features with their full name, unit, variable name, and, when relevant, their definition and their source.

The building of interest

1. Area of the building's footprint. Unit: squared meter. Variable name: `FootprintArea`
2. Perimeter of the building's footprint. Unit: meter. Variable name: `Perimeter`
3. Anisotropy index. Definition: the ratio between the area of the building footprint and the area of the circumscribed circle. Unit: $x \in [0, 1]$. Source: [1]. Variable name: `Phi`
4. Length of the longest axis of the building footprint. Definition: Axis is defined as a diameter of minimal circumscribed circle around the convex hull. Unit: meter. Source: [2]. Variable name: `LongestAxisLength`
5. Elongation of the minimum bounding box around the building footprint. Unit: $x \in [0, 1]$. Source: [2]. Variable name: `Elongation`
6. Convexity of the footprint. Definition: Area of the footprint divided by the area of the convex hull around the footprint. Unit: $x \in [0, 1]$. Source: [2]. Variable name: `Convexity`
7. Orientation of the footprint. Definition: orientation of the longext axis of bounding rectangle in range 0 - 45. It captures the deviation of orientation from cardinal directions. Unit: degree. Source: [2]. Variable name: `Orientation`
8. Number of corners of the footprint. Unit: count. Source: [2]. Variable name: `Corners`
9. Number of buildings directly adjacent to the building. Unit: count. Variable name: `CountTouches`
10. Length of wall shared with other buildings. Unit: meters. Variable name: `SharedWallLength`
11. Number of buildings in the block that the building is part of. Unit: count. Variable name: `BlockLength`

Blocks

12. Average footprint area of buildings in the block. Unit: squared meter. Variable name: `AvBlockFootprintArea`
13. Standard deviation of footprint areas of buildings in the block. Unit: squared meter. Variable name: `StdBlockFootprintArea`
14. Total footprint of the block. Unit: squared meters. Variable name: `BlockTotalFootprintArea`
15. Total perimeter of the block. Unit: meters. Variable name: `BlockPerimeter`

16. Length of the longest axis of whole block footprint. Unit: meters. Variable name: `BlockLongestAxisLength`
17. Elongation of the minimum bounding box around the whole block footprint. Unit: $x \in [0, 1]$. Variable name: `BlockElongation`
18. Convexity of the whole block footprint. Unit: $x \in [0, 1]$. Variable name: `BlockConvexity`
19. Orientation of the whole block footprint. Unit: degree. Variable name: `BlockOrientation`
20. Number of corners of the whole block footprint. Unit: count. Variable name: `BlockCorners`

<u>Buildings & blocks within 50 m</u>

21. Standard deviation blocks' orientation within 50 m around the building. Unit: degrees. Variable name: `std_block orientation_within_buffer_50`,
22. Average blocks' orientation within 50 m around the building. Unit: degrees. Variable name: `av_block_orientation_within_buffer_50`
23. Average individual building footprint area in blocks within 50 m around the building. Unit: squared meters. Variable name: `av_block_av_footprint_area_within_buffer_50`
24. Standard deviation block total footprint area within 50 m around the building. Unit: squared meters. Unit: Variable name: `std_block_footprint_area_within_buffer_50`
25. Average of the block total footprint area within 50 m around the building. Unit: squared meters. Variable name: `av_block_footprint_area_within_buffer_50`
26. Standard deviation of building count in blocks within 50 m around the building. Unit: count. Variable name: `std_block_length_within_buffer_50`
27. Average building count in blocks within 50 m around the building. Unit: count. Variable name: `av_block_length_within_buffer_50`
28. Number of blocks within 50 m around the building. Unit: count. Variable name: `blocks_within_buffer_50`
29. Standard deviation of building footprints within 50 m around the building. Unit: $x \in [0, 1]$. Variable name: `std_orientation_within_buffer_50`
30. Average orientation of building footprints within 50 m around the building. Unit: $x \in [0, 1]$. Variable name: `av_orientation_within_buffer_50`
31. Standard deviation of the convexity of building footprints within 50 m around the building. Unit: $x \in [0, 1]$. Variable name: `std_convexity_within_buffer_50`
32. Average convexity of building footprints within 50 m around the building. Unit: $x \in [0, 1]$. Variable name: `av_convexity_within_buffer_50`
33. Standard deviation of the elongation of buildings footprints within 50 m around the building. Unit: $x \in [0, 1]$. Variable name: `std_elongation_within_buffer_50`

34. Average elongation of buildings footprints within 50 m around the building. Unit: $x \in [0,1]$. Variable name: `av_elongation_within_buffer_50`

35. Standard deviation of building footprints area within 50 m around the building. Unit: squared meters. Variable name: `std_footprint_area_within_buffer_50`

36. Average building footprints area within 50 m around the building. Unit: squared meters. Variable name: `av_footprint_area_within_buffer_50`

37. Total building footprints area within 50 m around the building. Unit: squared meters. Variable name: `total_ft_area_within_buffer_50`

38. Number of buildings within 50 m around the building. Unit: counts. Variable name: `buildings_within_buffer_50`

    Buildings & blocks within 200 m

39. Standard deviation blocks' orientation within 200 m around the building. Unit: degrees. Variable name: `std_block_orientation_within_buffer_200`

40. Average blocks' orientation within 200 m around the building. Unit: degrees. Variable name: `av_block_orientation_within_buffer_200`

41. Average individual building footprint area in blocks within 200 m around the building. Unit: squared meters. Variable name: `av_block_av_footprint_area_within_buffer_200`

42. Standard deviation block total footprint area within 200 m around the building. Unit: squared meters. Variable name: `std_block_footprint_area_within_buffer_200`

43. Average of the block total footprint area within 200 m around the building. Unit: squared meters. Variable name: `av_block_footprint_area_within_buffer_200`

44. Average building count in blocks within 200 m around the building. Unit: count. Variable name: `std_block_length_within_buffer_200`

45. Average building count in blocks within 200 m around the building. Variable name: `av_block_length_within_buffer_200`

46. Number of blocks within 200 m around the building. Unit: count. Variable name: `blocks_within_buffer_200`

47. Standard deviation of building footprints within 200 m around the building. Unit: $x \in [0,1]$. Variable name: `std_orientation_within_buffer_200`

48. Average orientation of building footprints within 200 m around the building. Unit: $x \in [0,1]$. Variable name: `av_orientation_within_buffer_200`

49. Standard deviation of the convexity of building footprints within 200 m around the building. Unit: $x \in [0,1]$. Variable name: `std_convexity_within_buffer_200`

50. Average convexity of building footprints within 200 m around the building. Unit: $x \in [0,1]$. Variable name: `av_convexity_within_buffer_200`

51. Standard deviation of the elongation of building footprints within 200 m around the building. Unit: $x \in [0,1]$. Variable name: `std_elongation_within_buffer_200`

52. Average elongation of buildings footprints within 200 m around the building. Unit: $x \in [0, 1]$. Variable name: `av_elongation_within_buffer_200`
53. Standard deviation of building footprints area within 200 m around the building. Unit: squared meters. Variable name: `std_footprint_area_within_buffer_200`
54. Average building footprints area within 200 m around the building. Unit: squared meters. Variable name: `av_footprint_area_within_buffer_200`
55. Total building footprints area within 200 m around the building. Unit: squared meters. Variable name: `total_ft_area_within_buffer_200`
56. Number of buildings within 200 m around the building. Unit: counts. Variable name: `buildings_within_buffer_200`

    Buildings & blocks within 500 m

57. Standard deviation blocks' orientation within 500 m around the building. Unit: degrees. Variable name: `std_block_orientation_within_buffer_500`
58. Average blocks' orientation within 500 m around the building. Unit: degrees.Variable name: `av_block_orientation_within_buffer_500`
59. Average individual building footprint area in blocks within 500 m around the building. Unit: squared meters. Variable name: `av_block_av_footprint_area_within_buffer_500`
60. Standard deviation block total footprint area within 500 m around the building. Unit: squared meters. Variable name: `std_block_footprint_area_within_buffer_500`
61. Variable name: Average of the block total footprint area within 500 m around the building. Unit: squared meters. `av_block_footprint_area_within_buffer_500`
62. Average building count in blocks within 500 m around the building. Unit: count. Variable name: `std_block_length_within_buffer_500`
63. Average building count in blocks within 500 m around the building. Unit: count. Variable name: `av_block_length_within_buffer_500`
64. Number of blocks within 500 m around the building. Unit: count. Variable name: `blocks_within_buffer_500`
65. Standard deviation of building footprints within 500 m around the building. Unit: $x \in [0, 1]$. Variable name: `std_orientation_within_buffer_500`
66. Average orientation of building footprints within 500 m around the building. Unit: $x \in [0, 1]$. Variable name: `av_orientation_within_buffer_500`
67. Standard deviation of the convexity of building footprints within 500 m around the building. Unit: $x \in [0, 1]$. Variable name: `std_convexity_within_buffer_500`
68. Average convexity of building footprints within 500 m around the building. Unit: $x \in [0, 1]$.Variable name: `av_convexity_within_buffer_500`
69. Variable name: Average elongation of buildings footprints within 500 m around the building. Unit: $x \in [0, 1]$. `std_elongation_within_buffer_500`
70. Average elongation of buildings footprints within 500 m around the building. Unit: $x \in [0, 1]$. Variable name: `av_elongation_within_buffer_500`

71. Standard deviation of building footprints area within 50 m around the building. Unit: squared meters. Variable name: `std_footprint_area_within_buffer_500`

72. Average building footprints area within 500 m around the building. Unit: squared meters. Variable name: `av_footprint_area_within_buffer_500`

73. Total building footprints area within 500 m around the building. Unit: squared meters. Variable name: `total_ft_area_within_buffer_500`

74. Number of buildings within 500 m around the building. Unit: counts. Variable name: `buildings_within_buffer_500`

    <u>Street & intersection, closest</u>

75. Local closeness centrality for the closest street to the building. Definition: Local closeness for a radius of 500 m around each node. Value for one edge/street are averages of the values at the two nodes/intersections. Unit: Variable name: Unit: $x \in [0, 1]$. Source: [2]. `street_closeness_500_closest_road`

76. Betweeness centrality of the closest street to the building. Unit: $x \in [0, 1]$. Source: [2]. Variable name: `street_betweeness_global_closest_road`

77. Global closeness centrality of the closest street to the building. Unit: $x \in [0, 1]$. Source: [2]. Variable name: `street_closeness_global_closest_road`

78. Openness of the closest street to building. Definition: proportion of the street where buildings are or not present on the sides of the street. Unit: $x \in [0, 1]$. Source: [2]. Variable name: `street_openness_closest_road`

79. Standard deviation of the width of the closest street to the building. Definition: Width is defined here as the average distance between buildings on both sides of the street. Unit: meters Source: [2]. Variable name: `street_width_std_closest_road`

80. Average width of the closest street to the building. Unit: meters Source: [2]. Variable name: `street_width_av_closest_road`

81. Length of the closest street to the building. Unit: meters. Variable name: `street_length_closest_road`

82. Distance between the building and the closest street. Unit: meters. Variable name: `distance_to_closest_road`

83. Distance between the building and the closest intersection. Unit: meters. Variable name: `distance_to_closest_intersection`

    <u>Streets & intersections within 50 m</u>

84. Average local closeness centrality of the streets intersecting a 50 m buffer around the centroid of the building. Note: 500 m radius for the closeness centrality. Unit: $x \in [0, 1]$. Source: [2]. Variable name: `street_closeness_500_av_inter_buffer_50`

85. Largest local closeness centrality of the streets intersecting a 50 m buffer around the centroid of the building. Note: 500 m radius for the closeness centrality. Unit: $x \in [0, 1]$. Source: [2]. Variable name: `street_closeness_500_max_inter_buffer_50`

86. Average betwenness centrality of the streets intersecting a 50 m buffer around the centroid of the building. Unit: $x \in [0, 1]$. Source: [2]. Variable name: `street_betweeness_global_av_inter_buffer_50`
87. Largest betwenness centrality of the streets intersecting a 50 m buffer around the centroid of the building. Unit: $x \in [0, 1]$. Source: [2]. Variable name: `street_betweeness_global_max_inter_buffer_50`
88. Standard deviation of the width of the streets intersecting a 50 m buffer around the centroid of the building. Unit: meters Source: [2]. Variable name: `street_width_std_inter_buffer_50`
89. Average width of the streets intersecting a 50 m buffer around the centroid of the building. Unit: meters Source: [2]. Variable name: `street_width_av_inter_buffer_50`
90. Total length of streets intersecting a 50 m buffer around the centroid of the building. Unit: meters. Variable name: `street_length_total_inter_buffer_50`
91. Standard deviation length of streets *within* a 50 m buffer around the centroid of the building. Unit: meters. Variable name: `street_length_std_within_buffer_50`
92. Average length of streets *within* a 50 m buffer around the centroid of the building. Unit: meters. Variable name: `street_length_av_within_buffer_50`
93. Total length of streets *within* a 50 m buffer around the centroid of the building. Unit: meters. Variable name: `street_length_total_within_buffer_50`
94. Intersection count *within* a 50 m buffer around the centroid of the building. Unit: count. Variable name: `intersection_count_within_buffer_50`

    <u>Streets & intersections within 200 m</u>

95. Average local closeness centrality of the streets intersecting a 200 m buffer around the centroid of the building. Note: 500 m radius for the closeness centrality. Unit: $x \in [0, 1]$. Source: [2]. Variable name: `street_closeness_500_av_inter_buffer_200`
96. Largest local closeness centrality of the streets intersecting a 200 m buffer around the centroid of the building. Note: 500 m radius for the closeness centrality. Unit: $x \in [0, 1]$. Source: [2]. Variable name: `street_closeness_500_max_inter_buffer_200`
97. Average betwenness centrality of the streets intersecting a 200 m buffer around the centroid of the building. Unit: $x \in [0, 1]$. Source: [2]. Variable name: `street_betweeness_global_av_inter_buffer_200`
98. Largest betwenness centrality of the streets intersecting a 200 m buffer around the centroid of the building. Unit: $x \in [0, 1]$. Source: [2]. Variable name: `street_betweeness_global_max_inter_buffer_200`
99. Standard deviation of the width of the streets intersecting a 200 m buffer around the centroid of the building. Unit: meters Source: [2]. Variable name: `street_width_std_inter_buffer_200`
100. Average width of the streets intersecting a 200 m buffer around the centroid of the building. Unit: meters Source: [2]. Variable name: `street_width_av_inter_buffer_200`

101. Total length of streets intersecting a 200 m buffer around the centroid of the building. Unit: meters. Variable name: `street_length_total_inter_buffer_200`

102. Standard deviation length of streets *within* a 200 m buffer around the centroid of the building. Unit: meters. Variable name: `street_length_std_within_buffer_200`

103. Average length of streets *within* a 200 m buffer around the centroid of the building. Unit: meters. Variable name: `street_length_av_within_buffer_200`

104. Total length of streets *within* a 200 m buffer around the centroid of the building. Unit: meters. Variable name: `street_length_total_within_buffer_200`

105. Intersection count *within* a 200 m buffer around the centroid of the building. Unit: count. Variable name: `intersection_count_within_buffer_200`

Streets & intersections within 500 m

106. Average local closeness centrality of the streets intersecting a 500 m buffer around the centroid of the building. Note: 500 m radius for the closeness centrality. Unit: $x \in [0, 1]$. Source: [2]. Variable name: `street_closeness_500_av_inter_buffer_500`

107. Largest local closeness centrality of the streets intersecting a 500 m buffer around the centroid of the building. Note: 500 m radius for the closeness centrality. Unit: $x \in [0, 1]$. Source: [2]. Variable name: `street_closeness_500_max_inter_buffer_500`

108. Average betwenness centrality of the streets intersecting a 500 m buffer around the centroid of the building. Unit: $x \in [0, 1]$. Source: [2]. Variable name: `street_betweeness_global_av_inter_buffer_500`

109. Largest betwenness centrality of the streets intersecting a 500 m buffer around the centroid of the building. Unit: $x \in [0, 1]$. Source: [2]. Variable name: `street_betweeness_global_max_inter_buffer_500`

110. Standard deviation of the width of the streets intersecting a 500 m buffer around the centroid of the building. Unit: meters Source: [2]. Variable name: `street_width_std_inter_buffer_500`

111. Average width of the streets intersecting a 500 m buffer around the centroid of the building. Unit: meters Source: [2]. Variable name: `street_width_av_inter_buffer_500`

112. Total length of streets intersecting a 500 m buffer around the centroid of the building. Unit: meters. Variable name: `street_length_total_inter_buffer_500`

113. Standard deviation length of streets *within* a 500 m buffer around the centroid of the building. Unit: meters. Variable name: `street_length_std_within_buffer_500`

114. Average length of streets *within* a 500 m buffer around the centroid of the building. Unit: meters. Variable name: `street_length_av_within_buffer_500`

115. Total length of streets *within* a 500 m buffer around the centroid of the building. Unit: meters. Variable name: `street_length_total_within_buffer_500`

116. Intersection count *within* a 500 m buffer around the centroid of the building. Unit: count. Variable name: `intersection_count_within_buffer_500`

Street-based block, own block

117. Anisotropy index of the street-based block in which the building is. Unit: Unit: $x \in [0, 1]$. Variable name: `street_based_block_phi`
118. Area of the street-based block in which the building is. Unit: squared meters. Variable name: `street_based_block_area`

Street-based blocks within 50 m

119. Standard deviation of the street-based blocks intersecting a 50 m buffer around the centroid of the building. Unit: degrees Variable name: `street_based_block_std_orientation_inter_buffer_50`
120. Standard deviation of the anisotropy index of the street-based blocks intersecting a 50 m buffer around the centroid of the building. Unit: Unit: $x \in [0, 1]$. Variable name: `street_based_block_std_phi_inter_buffer_50`
121. Average anisotropy index of the street-based blocks intersecting a 50 m buffer around the centroid of the building. Unit: Unit: $x \in [0, 1]$. Variable name: `street_based_block_av_phi_inter_buffer_50`
122. Standard deviation of the area of the street-based blocks intersecting a 50 m buffer around the centroid of the building. Unit: squared meters. Variable name: `street_based_block_std_area_inter_buffer_50`
123. Average area of the street-based blocks intersecting a 50 m buffer around the centroid of the building. Unit: squared meters. Variable name: `street_based_block_av_area_inter_buffer_50`
124. Number of the street-based blocks intersecting a 50 m buffer around the centroid of the building. Unit: count Variable name: `street_based_block_number_inter_buffer_50`

Street-based blocks within 200 m

125. Standard deviation of the street-based blocks intersecting a 200 m buffer around the centroid of the building. Unit: degrees Variable name: `street_based_block_std_orientation_inter_buffer_200`
126. Standard deviation of the anisotropy index of the street-based blocks intersecting a 200 m buffer around the centroid of the building. Unit: Unit: $x \in [0, 1]$. Variable name: `street_based_block_std_phi_inter_buffer_200`
127. Average anisotropy index of the street-based blocks intersecting a 200 m buffer around the centroid of the building. Unit: Unit: $x \in [0, 1]$. Variable name: `street_based_block_av_phi_inter_buffer_200`
128. Standard deviation of the area of the street-based blocks intersecting a 200 m buffer around the centroid of the building. Unit: squared meters. Variable name: `street_based_block_std_area_inter_buffer_200`
129. Average area of the street-based blocks intersecting a 200 m buffer around the centroid of the building. Unit: squared meters. Variable name: `street_based_block_av_area_inter_buffer_200`

130. Number of the street-based blocks intersecting a 200 m buffer around the centroid of the building. Unit: count Variable name: `street_based_block_number_inter_buffer_200`

<u>Street-based blocks within 500 m</u>

131. Standard deviation of the street-based blocks intersecting a 500 m buffer around the centroid of the building. Unit: degrees Variable name: `street_based_block_std_orientation_inter_buffer_500`

132. Standard deviation of the anisotropy index of the street-based blocks intersecting a 500 m buffer around the centroid of the building. Unit: Unit: $x \in [0,1]$. Variable name: `street_based_block_std_phi_inter_buffer_500`

133. Average anisotropy index of the street-based blocks intersecting a 500 m buffer around the centroid of the building. Unit: Unit: $x \in [0,1]$. Variable name: `street_based_block_av_phi_inter_buffer_500`

134. Standard deviation of the area of the street-based blocks intersecting a 500 m buffer around the centroid of the building. Unit: squared meters. Variable name: `street_based_block_std_area_inter_buffer_500`

135. Average area of the street-based blocks intersecting a 500 m buffer around the centroid of the building. Unit: squared meters. Variable name: `street_based_block_av_area_inter_buffer_500`

136. Number of the street-based blocks intersecting a 500 m buffer around the centroid of the building. Unit: count Variable name: `street_based_block_number_inter_buffer_500`

<u>City level</u>

137. Total area of the city. Definition: area with the GDAM administrative boundary. Unit: squared meters. Variable name: `area_city`

138. Anisotropy index of the boundary of the city. Unit: $x \in [0,1]$. Variable name: `phi_city`

139. Totally number of buildings in the city. Unit: count. Variable name: `total_buildings_city`

140. Average building footprint area in the city. Unit: squared meters. Variable name: `av_building_footprint_city`

141. Standard deviation of the building footprints area in the city. Unit: squared meters. Variable name: `std_building_footprint_city`

142. Number of detached buildings in the city. Unit: count. Variable name: `num_detached_buildings`

143. Number of blocks from 2 to 5 buildings in the city. Unit: count. Variable name: `block_2_to_5`

144. Number of blocks from 6 to 10 buildings in the city. Unit: count. Variable name: `block_6_to_10`

145. Number of blocks from 11 to 20 buildings in the city. Unit: count. Variable name: `block_11_to_20`

146. Number of blocks above 20 buildings in the city. Unit: count. Variable name: `block_20+`

147. Total intersections count in the city. Unit: count. Variable name: `total_intersection_city`

148. Total street length in the city. Unit: meters. Variable name: `total_length_street_city`

149. Average street length in city. Unit: meters. Variable name: `av_length_street_city`

150. Number of street based blocks in the city. Unit: count. Variable name: `total_number_block_city`

151. Average area street-based blocks in the city. Unit: squared meters. Variable name: `av_area_block_city`

152. Standard deviation of the street-based blocks in the city. Unit: squared meters. Variable name: `std_area_block_city`

References

1. Louf R, Barthelemy M. A typology of street patterns. Journal of The Royal Society Interface. 2014;11(101):20140924.

2. Fleischmann M. MOMEPY: Urban morphology measuring toolkit. Journal of Open Source Software. 2019;4(43):1807.
