# Supplementary material for: Learning from urban form to predict building heights
Source: PLoS One. 2020 Dec 9;15(12):e0242010. doi: 10.1371/journal.pone.0242010 (PMC7725312; doi:10.1371/journal.pone.0242010)
Supplement: S4 Appendix — This appendix describes the details of the machine learning experimental procedure. The algorithms were trained on 32 CPU cores and 128GB of RAM, using the high performance computing cluster of the Potsdam Institute for Climate Impact Research. (PDF) [file pone.0242010.s004.pdf]

S4 Appendix. Machine learning experimental procedure. This appendix describes the details of the machine learning experimental procedure. The algorithms were trained on 32 CPU cores and 128GB of RAM, using the high performance computing cluster of the Potsdam Institute for Climate Impact Research.

- *Final data sets preparation.* The four folds for the cross-validation are the five French urban areas, Italy, and two parts of the Netherlands. Brandenburg is kept for testing, and Berlin is added to the training data of *Experiment 2*, but not used for the cross-validation (in order not to use local German data for training the parameters, but only data from other countries).

  We cut the Netherlands between the Northern and Southern parts, both to try have differences in the folds despite some adjacent regions and to balance the number of observations. The Northern part is composed of the regions of Groningen, Friesland, Drenthe, Overijssel, Flevoland, Gelderland, and Noord-Holland. The Southern part is composed of the regions of Utrecht, Zuid-Holland, Zeeland, Noord-Brabant, Limburg.

  The number of data points were initially for France n_{fr} = 772,776, for Italy n_{it} = 574,582, for the Netherlands n_{nl} = 9,170,167, for Berlin n_{ber} = 615,310 and for Brandenburg n_{bra} = 2,278,244.

  We removed null values from bugs or missing values in the original datasets. This amounted to 4.5% in France, 0% in Italy, 2% in the Netherlands, <1% in Brandenburg and Berlin.

  We then removed buildings below two meters and with a footprint below 10 m^2, because these represent small structures unlikely to be livable buildings, and are of lesser interest in the context of our study.

  Final values are for the five French urban areas n_{fr} = 654,881, for Italy n_{it} = 534,483, for two parts of the Netherlands respectively n_{nl1} = 3,952,009 and n_{nl2} = 3,729,484, for Berlin n_{ber} = 473,069 and for Brandenburg n_{bra} = 2,127,956.

- *Comparison between learning algorithms.* We compared the performance of a baseline median height, a linear regression algorithm, a random forest algorithm and a gradient boosting algorithm. For the linear regression and the random forest, we used the Python library `scikit-learn` [1], and for the gradient boosting the Python library `XGBoost` [2].

  For the baseline median height, we computed the median of the empirical heights distribution of each set, and used this same value for all data points in the error metrics. This simple model provides a benchmark, although such a model has no practical relevance.

  The linear regression was fitted in the standard implementation.

  For the random forest, we used the following hyper-parameters:

  – Number of trees (`n_estimators`): 450. We tried to increase up to 700 but this did not significantly improved the performance.
  – Number of features to consider at every split (`max_features`: squared
  – Maximum depth of a tree (`max_depth`): 60
  – Minimum number of samples required to split a node (`min_samples_split`): 5

  - Minimum number of samples required at each leaf node (`min_samples_leaf`): 4

  As results were consistent but XGBoost performed better than the random forest on premilimary experiments, we performed extensive grid search only on XGBoost.

- *Hyperparameters tuning.* For XGBoost, hyperparameters of models were tuned through a randomized grid-search with cross-validation [3]. The randomly generated grid that we tested is not exhaustive, but this reduces drastically the number of fits needed, while the method has proven empirically to be able to find often close to best hyperparameter values [3]. We used a four-fold cross-validation with 500 random combinations of 7 parameters, resulting in 2000 fits. At this stage, the number of gradient boosted trees (i.e. equivalent to number of boosting epochs) was limited to 100.

  Specifically, we used the following parameters and values:

  - Loss function (`loss`): regression with squared loss (`reg:squarederror`), regression with squared log loss (`reg:squaredlogerror`)
  - Learning rate (`learning_rate`): 0.01, 0.02, 0.03, 0.04, 0.05, 0.06, 0.07, 0.08, 0.09, 0.1
  - Maximum depth of a tree (`max_depth`): 3, 4, 5, 6, 7, 8, 9, 10, 11, 12, 13, 14, 15
  - Subsample ratio of the training instances (`subsample`): 0.5, 0.6, 0.7, 0.8, 0.9, 1
  - Subsample ratio of columns when constructing each tree (`colsample_bytree`): 0.5, 0.6, 0.7, 0.8, 0.9, 1
  - Minimum loss reduction required to make a further partition on a leaf node of the tree (`gamma`): 0, 5.55, 11.11, 16.66, 22.22, 27.77, 33.33, 38.88, 44.44, 50.0
  - Minimum sum of instance weight needed in a child (`min_child_weight`): 1, 5, 10, 15, 20, 25, 30, 35, 40, 45, 50

  We selected the final set of hyperparameters for XGBoost based on the best mean absolute error, averaged over the four folds. The best model achieved a MAE of 1.48m. The hyper-parameters values of the final model are:

  - Loss function: regression with squared loss
  - Learning rate: 0.05
  - Maximum depth of a tree: 15
  - Subsample ratio of the training instances: 1
  - Subsample ratio of columns when constructing each tree: 0.5
  - Minimum loss reduction required to make a further partition on a leaf node of the tree: 38.9
  - Minimum sum of instance weight needed in a child: 50

  For this best settings, we then optimized the early stopping behavior of the model. For this, we searched a eighth parameter, the number of gradient boosted trees (equivalent to number of boosting epochs, variable name: `n_estimator`). We retrieved the learning curves and picked the number of epochs where the minimum validation error was attained, before rising again due to overfitting. The best value for `n_estimator` was 45 for Italy, 334 for France, 246 for the first Dutch fold, and 198 for the second Dutch fold. We chose to set the value of `n_estimator` to 250, which is close to the average of the three highest number of epochs (259).

References

1. Pedregosa F, Varoquaux G, Gramfort A, Michel V, Thirion B, Grisel O, et al. Scikit-learn: Machine learning in Python. the Journal of machine Learning research. 2011;12:2825–2830.

2. Chen T, Guestrin C. Xgboost: A scalable tree boosting system. In: Proceedings of the 22nd acm sigkdd international conference on knowledge discovery and data mining; 2016. p. 785–794.

3. Bergstra J, Bengio Y. Random search for hyper-parameter optimization. The Journal of Machine Learning Research. 2012;13(1):281–305.
