# Supplementary material for: Learning from urban form to predict building heights
Source: PLoS One. 2020 Dec 9;15(12):e0242010. doi: 10.1371/journal.pone.0242010 (PMC7725312; doi:10.1371/journal.pone.0242010)
Supplement: S5 Appendix — (PDF) [file pone.0242010.s005.pdf]

S5 Appendix. Experiment with artificially balanced training set. To test the hypothesis that the poor predictions of the model on high buildings were due to statistical reasons – namely that high buildings above 10 m are much less than smaller buildings – we removed a large fraction of small buildings the training data.

We used the four bins sizes [2,5(, [5,10(, [10,15(, [15,inf(and retrieved for all training areas the number of buildings in the highest bin [15,inf(. Then, we sampled these amounts of buildings from the two medium groups, and 2/3 of them from the bin [2,5(because the range was smaller (3 m instead of 5 m). There were 32,954 data points above 15 m in France, 24,803 in Netherlands 1, 26,276 in Netherlands 2, and 5,380 in Italy. In total, the training set had 446,309 data points.

We tested this set-up for predicting on Brandenburg. The MAE of the model increased compared to *Experiment 1*, with 1.99 m here. This did not help improve the prediction of buildings in the [15,inf(either, as less than one percent were predicted well within a two-meter error range.
