# Supplementary material for: Learning from urban form to predict building heights
Source: PLoS One. 2020 Dec 9;15(12):e0242010. doi: 10.1371/journal.pone.0242010 (PMC7725312; doi:10.1371/journal.pone.0242010)
Supplement: S6 Appendix — (PDF) [file pone.0242010.s006.pdf]

S6 Appendix. Sensitivity analysis: specialized rural model. There are several reasons why a model focusing specifically on small and medium-sized towns in rural areas could be useful. Firstly, small- and medium-sized cities could benefit most from the data, as 3D models are usually created for larger cities. Secondly, they have fewer high buildings and thus less variability. Third, small cities may have a simpler urban morphology. Therefore, it could be possible to obtain a more accurate model specialised in predicting the height of buildings in small and medium-sized towns.

To test the hypothesis that such a specialised model would have a better performance, we removed all cities with a number of buildings above a threshold from the training and test set. The test is performed only for Brandenburg.

We chose 50,000 and 20,000 buildings as thresholds, and compared both to a case with the full training set. The former threshold represents slightly more buildings than the city of Potsdam. Potsdam has a 3D model, but this the case of few cities of that size and most of them do not have one, so our model is relevant for most cities of this size. The latter threshold is slightly lower than the city of Udine in Italy. Note that this thresholding approach could still keep certain small cities in the immediate surroundings of a large city.

With a 50,000 threshold, the number of data points in the training set becomes 8,157,896 for *Experiment 1* and 8,200,455 for *Experiment 2*. The test set has 1,923,504 data points. With a 20,000 threshold, the number of data points training set becomes for 4,323,269 *Experiment 1* and 4,358,035 for *Experiment 2*. The test set has 1,570,261 data points. The full training sets have 8,870,857 and 8,910,112 data points for *Experiment 1* and *2* respectively.

We find that a specialized model does not substantially improve the predictions of the full model (see S11 Table). Taking the same test set, there is no performance gain for *Experiment 1* for both thresholds. For *Experiment 2*, there is slight decrease (−4 cm) for the high threshold, but a slight increase for low threshold (+3 cm). These results indicate that the full model seems able to differentiate well across city size already, maybe through the city level features.
