# Supplementary material for: Learning from urban form to predict building heights
Source: PLoS One. 2020 Dec 9;15(12):e0242010. doi: 10.1371/journal.pone.0242010 (PMC7725312; doi:10.1371/journal.pone.0242010)
Supplement: S7 Appendix — (PDF) [file pone.0242010.s007.pdf]

S7 Appendix. Sensitivity analysis: discarding high outliers. As the model in its current form performs better on lower buildings than higher ones, it may be relevant to train a model on low buildings only, by removing highest buildings from the datasets. We tried two set-ups: in the first one, we removed buildings above a given height in both the training and the test set; in the second, we removed the buildings only from the training set. We tried both set-ups for Brandenburg, for *Experiments 1* and *2*.

We used three thresholds: 40 m, 30 m and 20 m. Above 40 m corresponds to high industrial buildings, skyscrapers, and high residential tower blocks. Above 30 m and 20 m would filter some other blocks and industrial buildings, as well as townhouses from the XIXth and early XXth centuries. We do not go below 20 m, as this would limit the variability in the dataset too much.

For *Experiment 1*, a threshold at 40 m reduces the number of data points in the training set to 8,868,489 and when applied to the test set, it reduces it to 1,922,836. A threshold at 30 m reduces the number of data points in the training set to 8,863,085 and when applied to the test set, it reduces it to 1,922,077. A threshold at 20 m reduces the number of data points in the training set to 8,831,587 and when applied to the test set, it reduces it to 1,917,987.

For *Experiment 2*, a threshold at 40 m reduces the number of data points in the training set to 8,911,031. A threshold at 30 m reduces the number of data points in the training set to 8,905,608. A threshold at 20 m reduces the number of data points in the training set to 8,874,017. For comparison, the values for the full training and test sets are 8,870,857 and 1,923,504.

Removing high outliers from both sets generates a small error decrease, while errors remain constant when the threshold is applied only to the training set (see S13 Table). With the lowest threshold at 20 m, the MAE decreases of 7 cm for *Experience 1*, and 3 cm for *Experience 2*. These small improvements do not seem to justify removing the outliers from the model, but this experiment should be reproduced with larger datasets.
