# Supplementary material for: Learning from urban form to predict building heights
Source: PLoS One. 2020 Dec 9;15(12):e0242010. doi: 10.1371/journal.pone.0242010 (PMC7725312; doi:10.1371/journal.pone.0242010)
Supplement: S8 Appendix — (PDF) [file pone.0242010.s008.pdf]

S8 Appendix. Spatial auto-correlation analysis. There are reasons to believe that some building heights are spatially auto-correlated. For example, in low-rise residential areas, many buildings have the same height. In such a case, our approach might not make use of all information available, and might be biased.

Therefore, we tested to which extent our model manages to capture the spatial auto-correlation. We computed a set-level measure of spatial auto-correlation, the global Moran's I. We used the library `PySAL` [1]. We computed the spatial weights matrix using k-nearest neighbors as distance metric, with $k = 4$. The Moran's I takes values between –1 and 1. A value close to 1 indicates a large level of clustering, a value close to –1 indicates a very regular dispersion, and 0 no spatial auto-correlation. These are statistical tests, therefore the p-value to assess their statistical significance is reported with the values in S15 Table.

We tested the spatial auto-correlation in both the output and the residuals. If no spatial auto-correlation is found, this would confirm the robustness of the method for these data. If there is spatial auto-correlation in the output but not in the residuals, this would suggest that the predictive features explain observed patterns. We additionally assessed the impact of our features of the urban morphology surrounding a building to account for spatial auto-correlation. Indeed, we can expect that the information at different buffer sizes implicitly captures some correlations. We computed the global Moran's I for additional model runs with the 8 features describing the building itself only, and compared these to the model runs with the full set of features.

Firstly, the results of the test show that there is spatial auto-correlation in buildings heights, both in the target distribution and the output, and both values are close when using the full set of features (see S15 Table). For Berlin, the spatial auto-correlation of the target heights is higher (0.64) than for Brandenburg (0.28), but the Moran's I of the output is within +/– 0.05, when the full set of features in used. We see this as an indication that the model reproduces relatively well the data. If the features on the surroundings are removed, the auto-correlation of the outputs decreases of about 0.17 to 0.30. This mismatch seems to show the role of the surroundings to reproduce the spatial auto-correlation in the data.

Secondly, there is some spatial auto-correlation in the residuals, but the value of the Moran's I is relatively low for three out of four experiments with the full set of features (between 0.20 and 0.26, only Berlin in Experiment 1 is 0.45). In three cases, the full set of feature provides an improvement over the building-only features. There is an especially large effect in Berlin for *Experiment 2*, with a decrease from 0.54 to 0.23. This is line with the excellent performance of this set-up in Berlin compared to other experiments in Berlin (see Table 4 and Fig 5. The effect is smaller for Brandenburg (0 to –0.06), but there is in general less spatial auto-correlation in Brandenburg (between 0.20 and 0.26 across all experiments).

Overall, this analysis indicates that the model with the full set of features, describing the surroundings of a building, is able to handle a reasonable part of the spatial auto-correlation in building heights. The analysis also shows that there is some margin of improvement to better capture the spatial auto-correlation with more advanced spatial methods. However, as the spatial auto-correlation seems to be very specific for different cities, including such approaches would also open up questions regarding the generalizability to contexts that are not included in the training set.

References

1. Rey SJ, Anselin L. PySAL: A Python library of spatial analytical methods. In: Handbook of applied spatial analysis. Springer; 2010. p. 175–193.
