# Supplementary material for: Learning from urban form to predict building heights
Source: PLoS One. 2020 Dec 9;15(12):e0242010. doi: 10.1371/journal.pone.0242010 (PMC7725312; doi:10.1371/journal.pone.0242010)
Supplement: S9 Appendix — (PDF) [file pone.0242010.s009.pdf]

S9 Appendix. Comparison building heights between OSM and the 3D model. We compared the building heights in OSM and in the respective 3D models for Berlin and Brandenburg.

For this, first we retrieved buildings where the tag `height` had a value in OSM. For Berlin, we found 9476 buildings with heights, which corresponds to about 2% of the 3D dataset for Berlin. There are 8784 buildings above 2 meters. For Brandenburg, we found only 2137 buildings, which corresponds to 0.1% of the 3D dataset. There were 1828 buildings above 2 meters.

Then, we joined OSM and the relevant 3D dataset based on the footprint polygons. We used the criterion *Intersection over Union* for the spatial joins. For this, we computed the ratio of the intersected area between two polygons over the combined area of the two polygons. We chose a conservative value of 0.8 for the matching the buildings, which led to large loses of buildings. We could match 1997 buildings for Berlin (21%) and 359 buildings for Brandenburg (17%). The large loss of buildings would be problematic if the data were to be use to train the model, as it drastically reduces the size of the training data set. The reasons for this is likely to be mismatches between the footprint definitions, as mentioned in the section Materials and Methods. More refined methods would be necessary to improve the matching.

The error between the two distributions on the samples is reasonable: the MAE is 2.62 m for Berlin and 3.12 m for Brandenburg. This confirms the relevance of our *Experiment 2*, and the noise in the OSM data is likely to be at least partly manageable for the model. The shape of the distribution for the OSM samples differ from the whole 3D models, and the OSM samples tend to have proportionally more high buildings. There were data points for all height groups, but the extent to which each sample covers the range of building types for each height is unsure. All this analysis is specific to Berlin and Brandenburg and does not have external validity for other regions.
