# Supplementary figures and images for: Learning from urban form to predict building heights
Source: PLoS One. 2020 Dec 9;15(12):e0242010. doi: 10.1371/journal.pone.0242010 (PMC7725312; doi:10.1371/journal.pone.0242010)

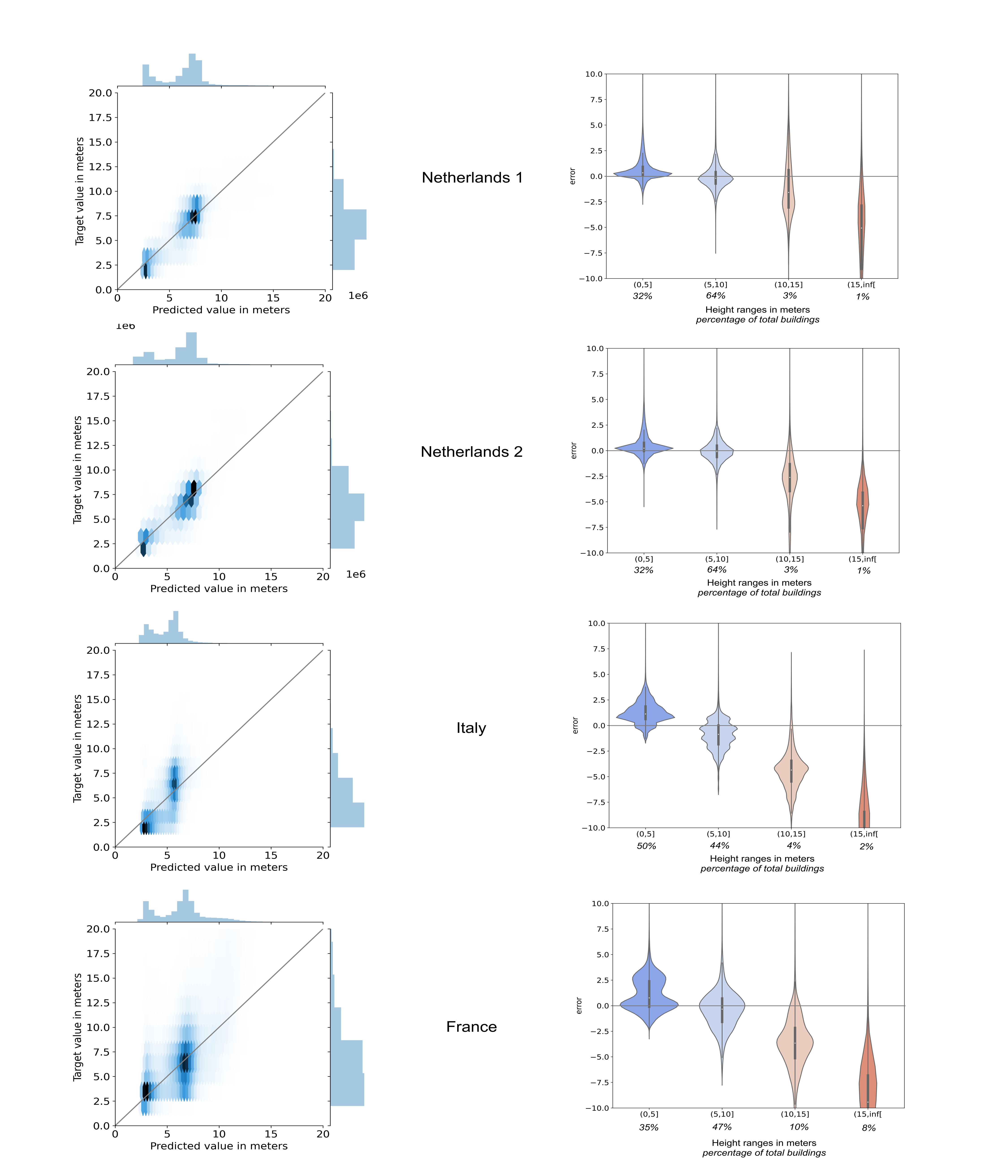

Supplement: S1 Fig — Left: for each fold, joint plot of predicted values over target values, both in meters. The intensity of the color of the bins represent the density of data points in the bin. On the thick diagonal grey line, points are perfectly predicted, and the light greys lines represent a + /− 2 meters error interval. Right: For each fold, error distribution of different target height ranges. (TIF) [file pone.0242010.s010.tif]

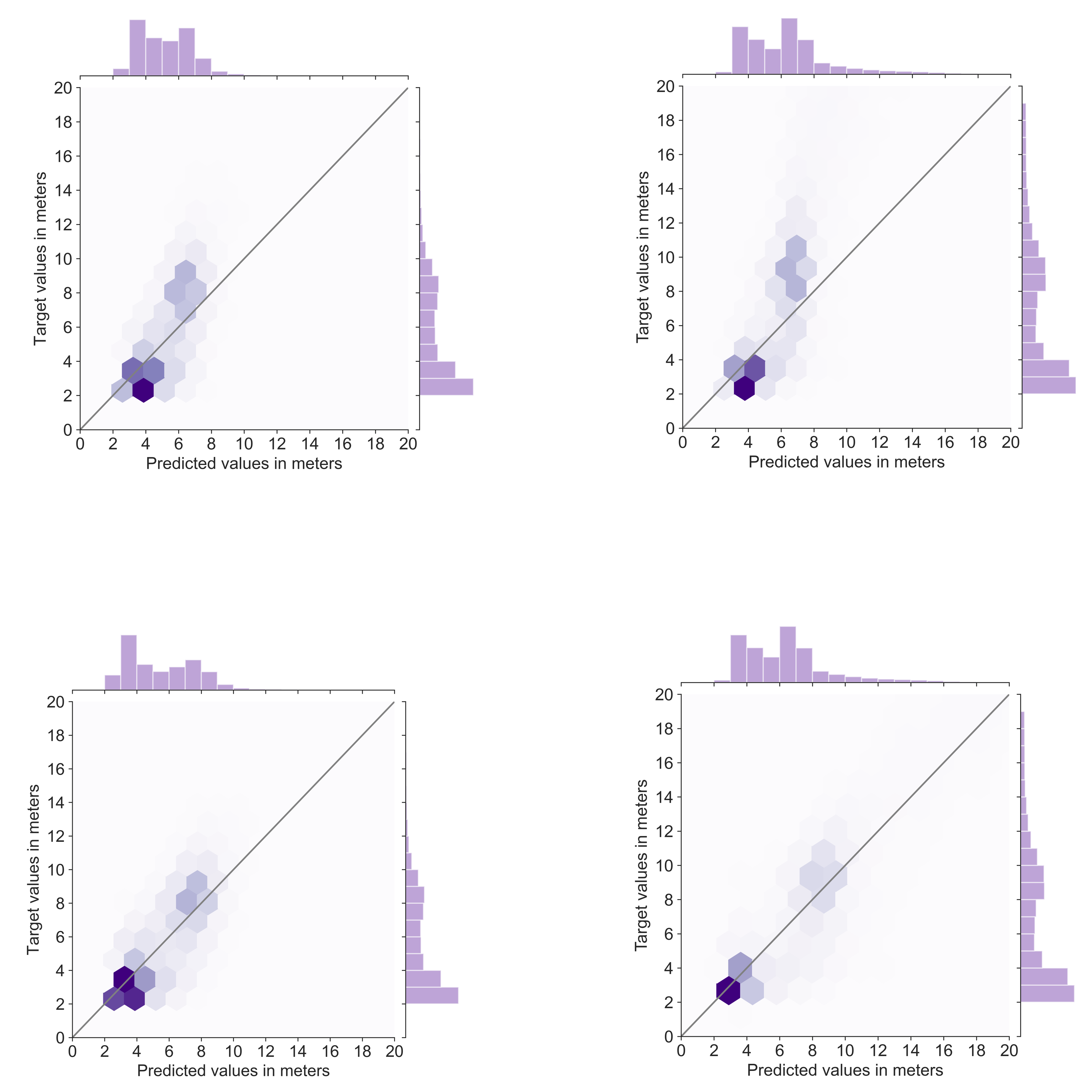

Supplement: S2 Fig — Jointplot of prediction errors for Experiment 1 (top) and Experiment 2 (bottom) for Brandenburg (left) and Berlin (right). (TIF) [file pone.0242010.s011.tif]

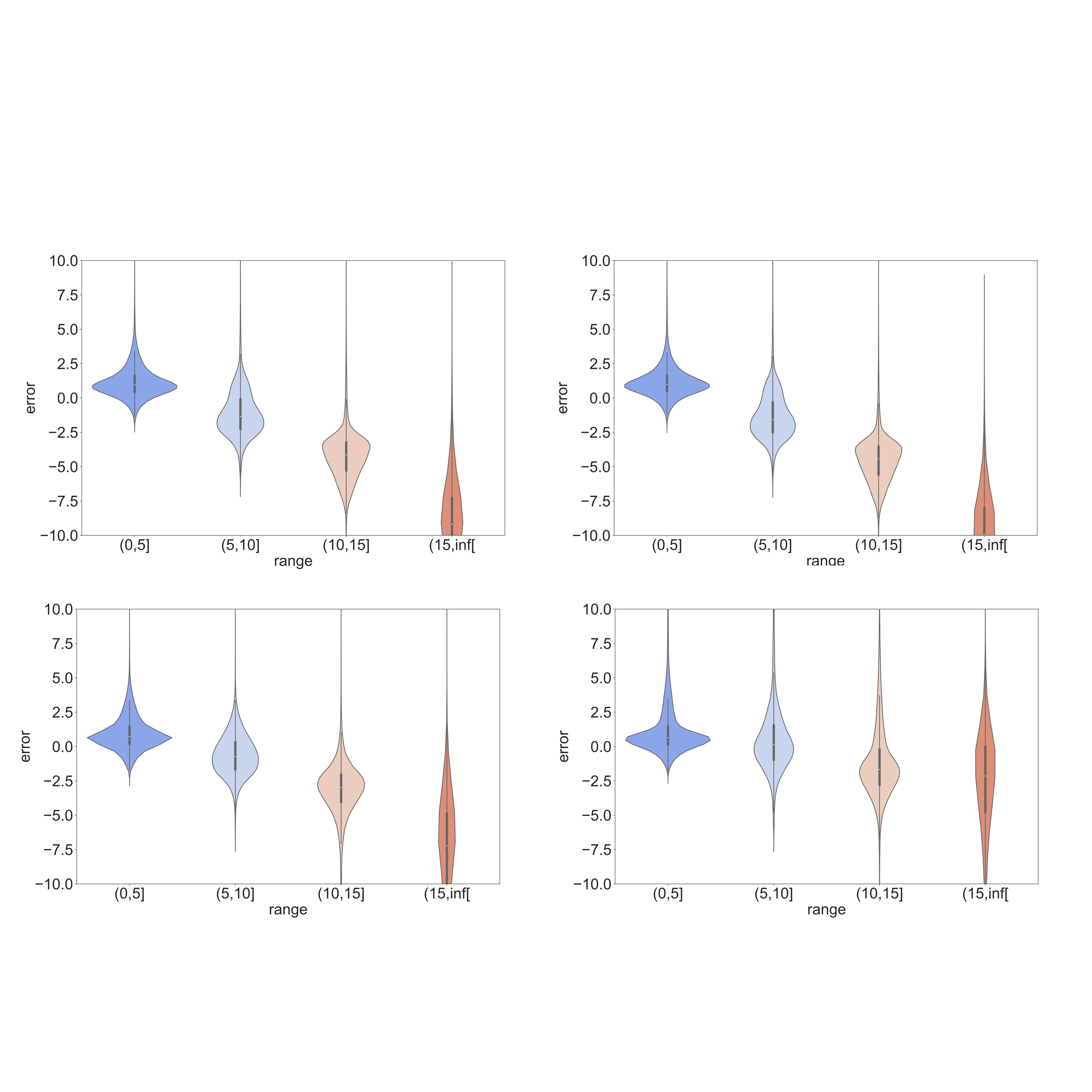

Supplement: S3 Fig — Violin plot of prediction errors by height ranges for Experiment 1 (top) and Experiment 2 (bottom) for Brandenburg (left) and Berlin (right). (TIF) [file pone.0242010.s012.tif]
