# Supplementary material for: Learning from urban form to predict building heights
Source: PLoS One. 2020 Dec 9;15(12):e0242010. doi: 10.1371/journal.pone.0242010 (PMC7725312; doi:10.1371/journal.pone.0242010)
Supplement: S1 Table — (PDF) [file pone.0242010.s013.pdf]

S1 Table. Feature importance test set

Feature	Gain	Relative
Brandenburg Experiment 2		
FootprintArea	15157	0.114
Perimeter	11942	0.089
LongestAxisLength	9000	0.067
BlockPerimeter	7091	0.053
av_block_length_within_buffer_500	6988	0.052
std_block_length_within_buffer_500	6637	0.049
BlockTotalFootprintArea	6242	0.046
BlockLongestAxisLength	2646	0.019
av_block_footprint_area_within_buffer_500	2315	0.017
distance_to_closest_road	2016	0.015
Brandenburg Experiment 1		
FootprintArea	12053	0.167
av_block_length_within_buffer_500	5281	0.073
BlockTotalFootprintArea	4623	0.064
Perimeter	3038	0.042
std_block_length_within_buffer_500	2958	0.041
av_block_footprint_area_within_buffer_500	1978	0.027
av_area_block_city	1167	0.016
AvBlockFootprintArea	1139	0.015
distance_to_closest_road	1073	0.014
street_length_total_within_buffer_500	950	0.013
