# Supplementary material for: Learning from urban form to predict building heights
Source: PLoS One. 2020 Dec 9;15(12):e0242010. doi: 10.1371/journal.pone.0242010 (PMC7725312; doi:10.1371/journal.pone.0242010)
Supplement: S2 Table — (PDF) [file pone.0242010.s014.pdf]

S2 Table. Results of a specialized rural model for Brandenburg. The table reports the mean absolute error in meters on the test set for each experiment and threshold. Thresholds correspond to the maximum number of building in cities that were included in the training and test sets. The results of this table should be compared vertically for a given test set.

Threshold on:	*Exp. 1: No local data*			*Exp. 2: Adding a 2%-sample*		
Test / Train	<20,000	<50,000	none	<20,000	<50,000	none
<20,000	1.71	–	–	1.51	–	–
<50,000	–	1.72	–	–	1.47	–
none	1.69	1.72	1.72	1.48	1.51	1.47
