# Supplementary material for: Learning from urban form to predict building heights
Source: PLoS One. 2020 Dec 9;15(12):e0242010. doi: 10.1371/journal.pone.0242010 (PMC7725312; doi:10.1371/journal.pone.0242010)
Supplement: S3 Table — The table reports the mean absolute error in meters on the test set for each experiment and threshold. Thresholds correspond to the maximum height of buildings included in the training and test sets. We report results for two set-ups: in the first one (diagonal), we remove buildings above a given height in both the training and the test set; in the second, we remove the buildings only from the training set (vertical column with ‘none’ for Test). The results of this table should be compared vertically for a given test set. (PDF) [file pone.0242010.s015.pdf]

S3 Table. Results of removing high outliers from the datasets, for Brandenburg. The table reports the mean absolute error in meters on the test set for each experiment and threshold. Thresholds correspond to the maximum height of buildings included in the training and test sets. We report results for two set-ups: in the first one (diagonal), we remove buildings above a given height in both the training and the test set; in the second, we remove the buildings only from the training set (vertical column with 'none' for Test). The results of this table should be compared vertically for a given test set.

Threshold on:	*Exp. 1: No local data*				*Exp. 2: Adding a 2%-sample*			
Train \ Test	20 m	30 m	40 m	none	20 m	30 m	40 m	none
20 m	1.65	–	–	1.71	1.44	–	–	1.50
30 m	–	1.69	–	1.72	–	1.47	–	1.51
40 m	–	–	1.70	1.72	–	–	1.49	1.51
none	1.71	1.72	1.72	1.72	1.50	1.51	1.51	1.47
