# Supplementary material for: Learning from urban form to predict building heights
Source: PLoS One. 2020 Dec 9;15(12):e0242010. doi: 10.1371/journal.pone.0242010 (PMC7725312; doi:10.1371/journal.pone.0242010)
Supplement: S4 Table — We report a set-level measure of spatial auto-correlation, the global Moran’s I, which is computed in Brandenburg and Berlin for the two experiments, with and without features describing the surroundings of a building. The Moran’s I is computed on both the residuals and the output of the model. Values for the target heights are reported for comparison. (PDF) [file pone.0242010.s016.pdf]

S4 Table. Moran's I of the model's outputs and residuals in various settings for Berlin and Brandenburg. We report a set-level measure of spatial auto-correlation, the global Moran's I, which is computed in Brandenburg and Berlin for the two experiments, with and without features describing the surroundings of a building. The Moran's I is computed on both the residuals and the output of the model. Values for the target heights are reported for comparison.

		Brandenburg		Berlin	
Experience	Surroundings	Output	Residuals	Output	Residuals
Exp. 1: No local data	with	0.34^{***}	0.26^{***}	0.64^{***}	0.45^{***}
	without	0.15^{***}	0.26^{***}	0.28^{***}	0.54^{***}
Exp. 2: Adding a 2%-sample	with	0.32^{***}	0.20^{***}	0.59^{***}	0.23^{***}
	without	0.15^{***}	0.26^{***}	0.29^{***}	0.54^{***}
Target heights		0.28^{***}		0.55^{***}	

The Moran's I takes values between -1 and 1. A value close to 1 indicates a large level of clustering, a value close to -1 indicates a very regular dispersion, and 0 no spatial auto-correlation. These are statistical tests, therefore the p-value to assess their statistical significance is reported: $^{*}p < 0.1$; $^{**}p < 0.05$; $^{***}p < 0.01$.
