# Supplementary material for: Learning from urban form to predict building heights
Source: PLoS One. 2020 Dec 9;15(12):e0242010. doi: 10.1371/journal.pone.0242010 (PMC7725312; doi:10.1371/journal.pone.0242010)
Supplement: S1 Code — (PDF) [file pone.0242010.s017.pdf]

S1 Code. Scripts. All the code used in this study, as well as a tutorial on how to replicate the experiments is available on the public repository `https://gitlab.pik-potsdam.de/nikolami/learning-from-urban-form-to-predict-building-heights`.
